# Coordination tuning of cobalt phosphates towards efficient water oxidation catalyst

**DOI:** 10.1038/ncomms9253

**Published:** 2015-09-14

**Authors:** Hyunah Kim, Jimin Park, Inchul Park, Kyoungsuk Jin, Sung Eun Jerng, Sun Hee Kim, Ki Tae Nam, Kisuk Kang

**Affiliations:** 1 Department of Materials Science and Engineering, Research Institute of Advanced Materials (RIAM), Seoul National University, 1 Gwanak-ro, Gwanak-gu, Seoul 151-742, Republic of Korea; 2 Center for Biomaterials, Korea Institute of Science and Technology, 5, Hwarang-ro 14-gil, Seongbuk-gu, Seoul 136-791, Republic of Korea; 3 Western Seoul Center, Korea Basic Science Institute (KBSI), 150, Bukahyeon ro, Seodaemun-gu, Seoul 120-140, Korea; 4 Center for Nanoparticle Research, Institute for Basic Science (IBS), Seoul National University, 1 Gwanak-ro, Gwanak-gu, Seoul 151-742, Republic of Korea

## Abstract

The development of efficient and stable water oxidation catalysts is necessary for the realization of practically viable water-splitting systems. Although extensive studies have focused on the metal-oxide catalysts, the effect of metal coordination on the catalytic ability remains still elusive. Here we select four cobalt-based phosphate catalysts with various cobalt- and phosphate-group coordination as a platform to better understand the catalytic activity of cobalt-based materials. Although they exhibit various catalytic activities and stabilities during water oxidation, Na_2_CoP_2_O_7_ with distorted cobalt tetrahedral geometry shows high activity comparable to that of amorphous cobalt phosphate under neutral conditions, along with high structural stability. First-principles calculations suggest that the surface reorganization by the pyrophosphate ligand induces a highly distorted tetrahedral geometry, where water molecules can favourably bind, resulting in a low overpotential (∼0.42 eV). Our findings emphasize the importance of local cobalt coordination in the catalysis and suggest the possible effect of polyanions on the water oxidation chemistry.

## Abstract (web summary)

The role of metal-coordination environment on the effectiveness of water oxidation catalysts is relatively unexplored. Here, the authors study a range of cobalt phosphate catalysts with varying coordination environments and observe high activity and stability for a distorted tetrahedral cobalt system.

## Graphical abstract

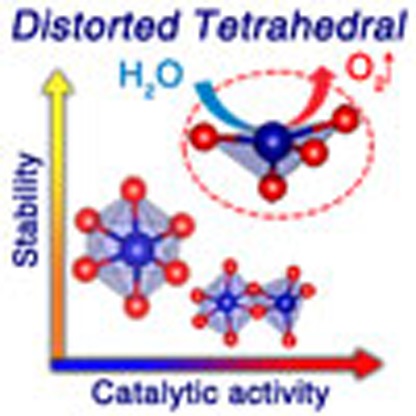

Splitting water into hydrogen and oxygen molecules via solar energy has been considered as one of the most environment-friendly ways to effectively use renewable energy resources from harvest to redistribution^1^,^2^,^3^,^4^,^5^,^6^. Although it has been studied for more than a half century, the inefficiency of the oxygen evolution reaction (OER) has not yet been resolved and is still regarded as a bottleneck in the integration of an overall water splitting system^1^,^2^,^3^,^4^,^5^,^6^,^7^,^8^. This inefficiency is primarily due to the rigid O–O double bond formation and the sluggish proton-coupled electron-transfer reactions^7^,^8^,^9^. Noble-metal catalysts, such as Pt, IrO_*x*_ and RuO_*x*_, exhibit outstanding OER catalytic activity; however, the high costs of these catalysts prohibit their practical use^10^,^11^,^12^,^13^,^14^. In this regard, the development of cost-effective, abundant-element-based and efficient OER catalysts is highly demanded.

Inspired from the Mn-based water oxidation complex in photosystem II that generates oxygen from water with an exceptionally high efficiency^15^,^16^,^17^, numerous OER catalysts have been synthesized and evaluated using relatively cheaper 3*d* transition metals (Mn, Fe, Co and Ni)^8^,^18^,^19^,^20^,^21^,^22^,^23^,^24^,^25^,^26^,^27^,^28^,^29^,^30^,^31^,^32^,^33^,^34^,^35^,^36^. In particular, cobalt-containing catalysts have been studied as promising OER catalysts because of their high catalytic activity for water oxidation, which is comparable to those of precious-metal-based catalysts^8^,^23^,^24^,^25^,^26^,^27^,^28^,^29^,^30^,^31^,^32^,^33^,^34^,^35^,^36^. Various cobalt-based oxides, perovskites and layered double hydroxides have been developed and examined via experimental and theoretical approaches^23^,^24^,^25^,^26^,^27^,^28^,^29^,^30^,^31^,^32^,^33^,^34^,^35^,^36^. Among these materials, cobalt phosphate (Co-Pi) prepared using electrodeposition exhibits one of the highest performances to date with efficient and stable catalytic performance^8^. The extraordinary performance of Co-Pi has cast fundamental questions on the correlation between cobalt chemistry and the OER mechanism, and has further promoted the exploration of other cobalt-based OER catalysts.

Although various cobalt-based materials have been investigated as OER catalysts, researches were carried out mostly on materials with specific cobalt local environments such as octahedral (*O*_h_) cobalt^8^,^26^,^27^,^28^,^32^,^33^,^34^. Even amorphous Co-Pi has been recently shown to adopt the local *O*_h_ coordination of cobalt^26^. Although the coordination and corresponding electronic structure of the transition metal in the catalysts is believed to greatly affect the OER activity^19^,^20^,^21^,^22^,^23^,^31^,^32^,^33^,^34^, there is a lack of systematic information relating the local cobalt coordination to the OER catalysis. Moreover, in practice, most of the cobalt-based materials reported thus far have been prone to transform into amorphous Co-Pi or cobalt-oxide-like phases from the surface under the typical pH and voltage regions where the OER proceeds^27^,^28^,^29^,^30^,^31^. This transformation makes it more difficult to apprehend the correlation between the structural cobalt geometry and the OER mechanisms, which has in turn retarded the development of new cobalt-based OER catalysts.

Transition metal phosphates have positive attributes in structural stability, especially in oxidative environments over oxides, and can adopt versatile structures with various orientations of the phosphate group present in the crystal^37^,^38^,^39^. As shown in Supplementary Table 1, whereas conventional oxide materials have cobalt *O*_h_ geometry with higher-symmetry crystal structures, phosphate/pyrophosphate-containing compounds have various cobalt geometries including tetrahedral (*T*_d_), trigonal bipyramidal (TBP) and *O*_h_, with lower-symmetry crystal structures due to the bulky phosphate/pyrophosphate ligands. Moreover, the flexible coordination of phosphate groups can stabilize the intermediate state of the transition metal by changing their local positions with ease, ensuring an effective redox change in the transition metal^20^,^40^. Indeed, our recent study using Mn_3_(PO_4_)_2_·3H_2_O as a OER catalyst demonstrated that the inherently unstable Mn(III) state is stabilized by the structural flexibility of the phosphate group^20^. Along with the case of Co-Pi^30^, it implies that the interplay between the transition metal and phosphate groups in the structure can also affect the catalytic activity, which, if clearly understood, would aid in the discovery of new efficient OER catalysts.

Herein, we explore the OER catalytic capabilities of four cobalt-based phosphate materials with various cobalt environments, Na_2_CoP_2_O_7_, NaCoPO_4_, Li_2_CoP_2_O_7_ and LiCoPO_4_, which are well known as cobalt redox-active electrode materials in rechargeable lithium or sodium batteries^41^,^42^,^43^,^44^,^45^. Except for LiCoPO_4_, other three materials have not been evaluated as OER catalysts. All of these materials have crystal structures in which cobalt polyhedrons are cross-linked by phosphate groups. The uniqueness of this selection of materials lies in the various local cobalt coordination, including *O*_h_, *T*_d_ and TBP configurations, with different networks between the cobalt polyhedron. These different cobalt environments provide a platform to better understand the catalytic activity of cobalt-based materials in the OER. Interestingly, among the four candidates, we find that Na_2_CoP_2_O_7_ with *T*_d_ geometry has excellent catalytic activity and high stability comparable to those of the well-known amorphous Co-Pi. Cyclic voltammetry (CV), high-resolution transmission electron microscopy (HR-TEM), X-ray photoelectron spectroscopy (XPS) and *in-situ* X-ray absorption near edge spectroscopy (XANES), scanning transmission electron microscopy energy-dispersive X-ray spectroscopy (STEM-EDX) and electron paramagnetic resonance (EPR) confirms the catalytic and structural stability of Na_2_CoP_2_O_7_. Theoretical calculations reveal that a unique OER pathway is present for Na_2_CoP_2_O_7_, which uses four coordinated cobalt atoms with low-energy barriers for the water-splitting reaction.

## Results

### Cobalt phosphates as a new group of OER catalysts

**Figure 1:**
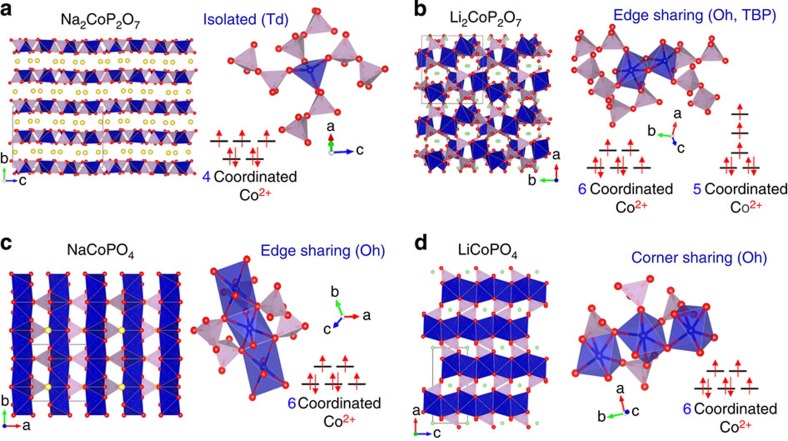
Crystal structure and cobalt crystal field of cobalt phosphate catalysts. Crystal structures of (**a**) Na_2_CoP_2_O_7_, (**b**) Li_2_CoP_2_O_7_, (**c**) NaCoPO_4_ and (**d**) LiCoPO_4_. The inset shows the local environment around the cobalt subunit (blue) and the spin state of the cobalt atom. The phosphate units, Na atoms and Li atoms are depicted in grey, yellow and green, respectively.

The Na_2_CoP_2_O_7_, Li_2_CoP_2_O_7_, NaCoPO_4_ and LiCoPO_4_ compounds were synthesized using conventional solid-state methods, as described previously^41^,^42^,^43^,^44^,^45^, and the X-ray diffraction (XRD) patterns of the synthesized compounds well matched those of previous reports without impurites^41^,^42^,^43^,^44^,^45^ (Supplementary Fig. 1). The local crystal structure and overall networks of the polyhedrons in these four materials are schematically shown in Fig. 1 based on Rietveld analysis of each material along with the lattice parameters (Supplementary Fig. 1 and Supplementary Table 2).

The phosphate and pyrophosphate groups in these four materials induced various cobalt coordination and local polyhedron networks. Specifically, Na_2_CoP_2_O_7_ and Li_2_CoP_2_O_7_ have *T*_d_ and mixed *O*_h_/TBP cobalt geometries, respectively, whereas the cobalt in NaCoPO_4_ and LiCoPO_4_ was only in the *O*_h_. *T*_d_ polyhedron with higher point symmetry in Na_2_CoP_2_O_7_ is not usual considering the fact that Co(II) is typically stabilized in *O*_h_ environments^27^,^28^,^29^. Moreover, the *T*_d_ polyhedrons are isolated by pyrophosphate groups, and with respect to the long-range ordering of the polyhedron the *T*_d_ polyhedra in Na_2_CoP_2_O_7_ form a two-dimensional layer via pyrophosphate groups, which is alternately stacked with sodium layers. Similarly, NaCoPO_4_ exhibited a two-dimensional nature; however, each edge-sharing *O*_h_ layer was cross-linked by a phosphate ligand. LiCoPO_4_ exhibited similar structures as NaCoPO_4_ in that two-dimensional chains of corner-sharing CoO_6_ were linked by a phosphate ligand. However, in Li_2_CoP_2_O_7_, Co_2_O_9_ subunits were interconnected through P_2_O_7_ groups to build a 3D framework structure. These various structural features differ from the well-studied edge-shared *O*_h_ CoO_6_ observed in most cobalt-based catalysts^8^,^26^,^27^,^28^,^32^,^33^,^34^.

### OER catalytic behaviour of the cobalt phosphate catalysts

**Figure 2:**
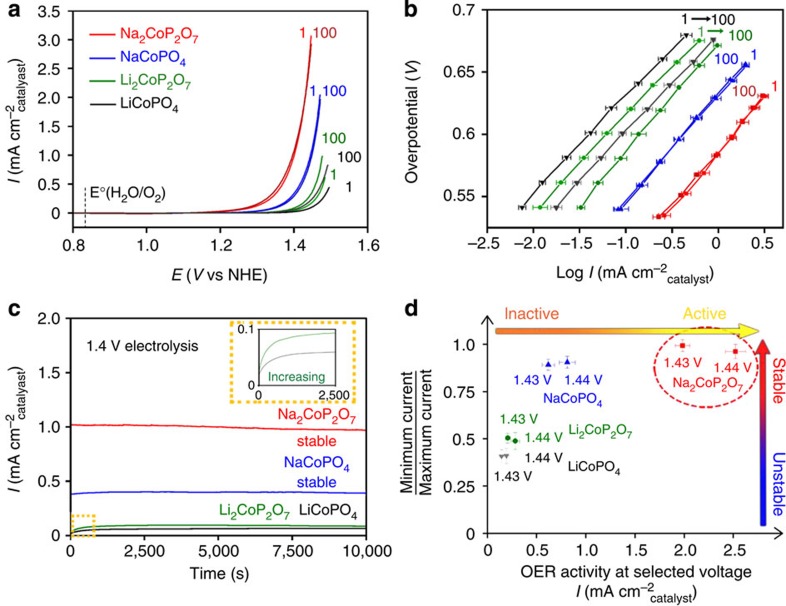
Oxygen evolution performances of cobalt phosphate catalysts. (**a**) CV curves and (**b**) Tafel plots of Na_2_CoP_2_O_7_, Li_2_CoP_2_O_7_, NaCoPO_4_ and LiCoPO_4_ for the 1st and 100th cycle from 0.7 to 1.5 V versus NHE. The thermodynamic potential for water oxidation is marked at 0.816 V versus NHE (pH 7.0). (**c**) Bulk electrolysis was performed under a constant potential of 1.4 V versus NHE. The inset displays the enlarged bulk electrolysis profiles of Li_2_CoP_2_O_7_ and LiCoPO_4_. (**d**) The electrochemical performances of Na_2_CoP_2_O_7_, Li_2_CoP_2_O_7_, NaCoPO_4_ and LiCoPO_4_ with respect to their catalytic activities and stabilities. OER current of 100th cycle at the selected voltage and the ratio of the minimum current to maximum current at the selected voltage during 100 cycles are depicted along the *x* axis and *y* axis, respectively. Error bars represent s.d. (*n*=5).

The water oxidation catalytic properties of Na_2_CoP_2_O_7_, Li_2_CoP_2_O_7_, NaCoPO_4_ and LiCoPO_4_ were evaluated by CV in 0.5 M sodium phosphate buffer at pH 7.0 using a previously reported method^21^,^23^. One hundred cycles of potential sweep from 0.7 to 1.5 V versus normal hydrogen electrode (NHE) were performed for each substrate (Supplementary Fig. 2). The 1st and 100th CV curves of Na_2_CoP_2_O_7_, Li_2_CoP_2_O_7_, NaCoPO_4_ and LiCoPO_4_ are shown in Fig. 2a. We observed that the catalytic activities increased in the series of LiCoPO_4_ <Li_2_CoP_2_O_7_ <NaCoPO_4_ <Na_2_CoP_2_O_7_. Na_2_CoP_2_O_7_ exhibited a current density of 2.62 mA cm^−2^ at 1.44 V versus NHE, which is significantly higher than those of NaCoPO_4_ (0.90 mA cm^−2^), Li_2_CoP_2_O_7_ (0.14 mA cm^−2^) and LiCoPO_4_ (0.08 mA cm^−2^) under identical conditions. The superior catalytic activity of Na_2_CoP_2_O_7_ compared with the other compounds was maintained even after 100 cycles. The activities of both Na_2_CoP_2_O_7_ and NaCoPO_4_ remained nearly unchanged during 100 cycles, whereas those of Li_2_CoP_2_O_7_ and LiCoPO_4_ slightly increased. The current densities of Li_2_CoP_2_O_7_ and LiCoPO_4_ changed from 0.14 to 0.29 mA cm^−2^ and from 0.08 to 0.19 mA cm^−2^ during 100 cycles at 1.44 V versus NHE, respectively. A similar current increase was previously observed in LiCoPO_4_ (ref. 27), which was explained by the phase transformation on the catalyst surface, as will be discussed in detail in the later section.

The observed catalytic trend was also confirmed for the Tafel and potentiostatic electrolysis (Fig. 2b,c and Supplementary Fig. 3). Figure 2b shows that the exchange current increases in the series of LiCoPO_4_ <Li_2_CoP_2_O_7_ <NaCoPO_4_ <Na_2_CoP_2_O_7_, where the exchange currents are high for NaCoPO_4_ and Na_2_CoP_2_O_7_ even after 100 cycles of CV scans. The Tafel slopes of Na_2_CoP_2_O_7_, NaCoPO_4_, Li_2_CoP_2_O_7_ and LiCoPO_4_ at the 100th cycle were calculated as 82, 85, 84 and 82 mV dec^−1^, respectively. Figure 2c indicates that the current densities of Na_2_CoP_2_O_7_ and NaCoPO_4_ in the potentiostatic electrolysis were stably maintained at a high value under constant potential 1.4 V versus NHE; in contrast, those of Li_2_CoP_2_O_7_ and LiCoPO_4_ were almost an order lower even though they slightly increased at the beginning of the electrolysis. It was found that the catalytic performance of Na_2_CoP_2_O_7_ was almost comparable to that of the electro-deposited Co-Pi in Supplementary Fig. 4. Despite the lower non-catalytic current, which is related to the number of redox-active cobalt ions or electrochemically active surface area^28^,^46^, the OER current of Na_2_CoP_2_O_7_ was higher than that of the electro-deposited Co-Pi at 1.4 V versus NHE (Supplementary Fig. 4). Comparison with β-CoOOH whose local structure is similar to that of Co-Pi exhibited similar trends (see details in the later part of the manuscript). However, it should be noted that, as Na_2_CoP_2_O_7_ was synthesized using a conventional solid-state reaction and not by electrodeposition, a further study for comparing the catalytic abilities of the two samples using a similar preparation method is needed.

In Fig. 2d, we summarized the electrochemical performance of four materials with respect to their catalytic activities and stabilities, that is, OER current after 100 cycles and the ratio of minimum to maximum current during the 100 cycles. A higher OER current along the *x* axis indicates a more active nature of the catalyst. Likewise, a higher current ratio close to unity along the *y* axis indicates better stability. As shown in Fig. 2d, Na_2_CoP_2_O_7_ exhibits the best catalytic activity and stability among the four materials. Moreover, further long-term stability tests for Na_2_CoP_2_O_7_ revealed that the catalytic current can be stably maintained during 500 cycles of CV scans and 12 h of bulk electrolysis (Supplementary Fig. 5). In the following, a more detailed investigation is focused primarily on Na_2_CoP_2_O_7_.

**Figure 3:**
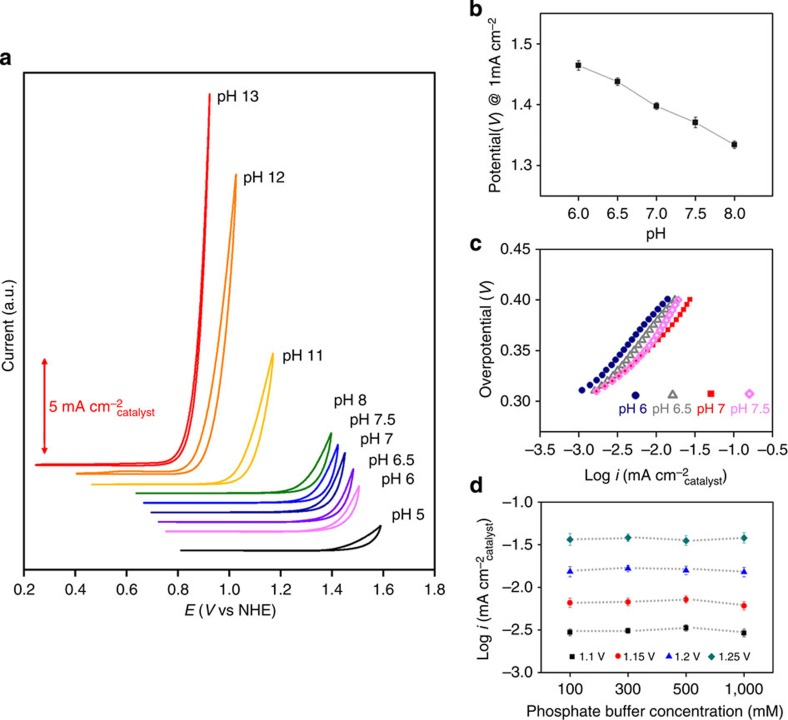
OER catalytic performances of Na_2_CoP_2_O_7_ at various pH and phosphate buffer concentrations. (**a**) CV curves for Na_2_CoP_2_O_7_ from pH 5 to pH 13. (**b**) pH-dependent potential change at a constant current density of 1 mA cm^−2^. (**c**) Tafel plots of Na_2_CoP_2_O_7_ from pH 6 to pH 7.5. (**d**) Phosphate buffer concentration dependency of the OER current at a constant applied potential (1.10, 1.15, 1.2 and 1.25 V versus NHE) at pH 7. Error bars represent s.d. (*n*=5).

For deeper investigation of the OER performance of Na_2_CoP_2_O_7_, CV curves of Na_2_CoP_2_O_7_ were recorded under various pH conditions from pH 5 to pH 13. As shown in Fig. 3a, the onset potential for Na_2_CoP_2_O_7_ shifted towards a more negative potential with an increase in pH. The catalytic current was stably maintained during the OER under basic condition, whereas degradation in the catalytic current was observed during the OER tests at pH 5 (Supplementary Fig. 6).

The OER kinetics of Na_2_CoP_2_O_7_ was examined by varying the pH and the phosphate concentration of the electrolytes (Fig. 3a). First, we investigated the pH-dependent potential change at a constant current density for understanding the dependence of the reaction rate on proton activity^47^,^48^,^49^. A plot between the pH and applied potential at constant current density of 1 mA cm^−2^ exhibited linearity from pH 6.0 to 8.0 and the slope was calculated as approximately −67 mV per  pH (Fig. 3b). The Tafel slopes for pH 6, 6.5 and 7.5 were measured as ∼80 mV dec^−1^, similar to the value at pH 7 (Fig. 3c). Then, the dependence of the reaction rate on the proton activity was derived by following equation^47^,^48^,^49^:


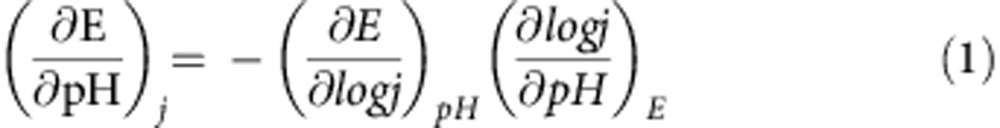


Substituting 
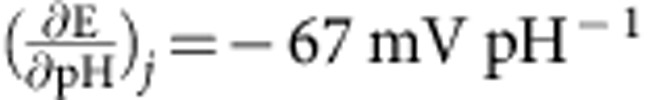
 and 
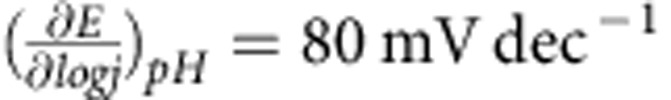
 into the equation (1) leads to 
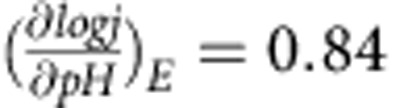
. This result indicated that the reaction rate follows an approximately inverse first-order dependence on the proton activity^47^,^48^,^49^. In addition, the dependence of the reaction rate on the phosphate-buffer strength was also examined by varying the buffer strength from 100 mM to 1 M. No change in the catalytic current depending on the buffer strength was found, indicating zero-order dependence on the phosphate-buffer strength (Fig. 3d)^47^,^48^.

Although the measured Tafel slopes were somewhat far from 2.3 RT F^−1^, typically 60∼80 mV dec^−1^ of Tafel slopes indicate that a one electron-transfer step is involved before rate determinant step (RDS)^48^,^49^. The exact reason for the higher Tafel values of our materials than 2.3 RT F^−1^ is unclear, but one possible explanation is that limitation in ion or electron transport might inflate the Tafel slope, as proposed in previous reports for LiCoPO_4_ and thick Co-Pi films^27^.

Although there is some deviation between the theoretical values and our experimental values, based on the Tafel and pH-dependent analysis, we can postulate that a one-proton/one-electron-involved equilibrium step may exist before the RDS in the OER mechanism of Na_2_CoP_2_O_7_ (refs 47, 48, 49). Considering the calculated RDS of Na_2_CoP_2_O_7_ was Co(IV)=O formation, as described in a later section, we could expect that equilibrium between Co(II) and Co(III) is the prior step before the RDS in the OER.

**Figure 4:**
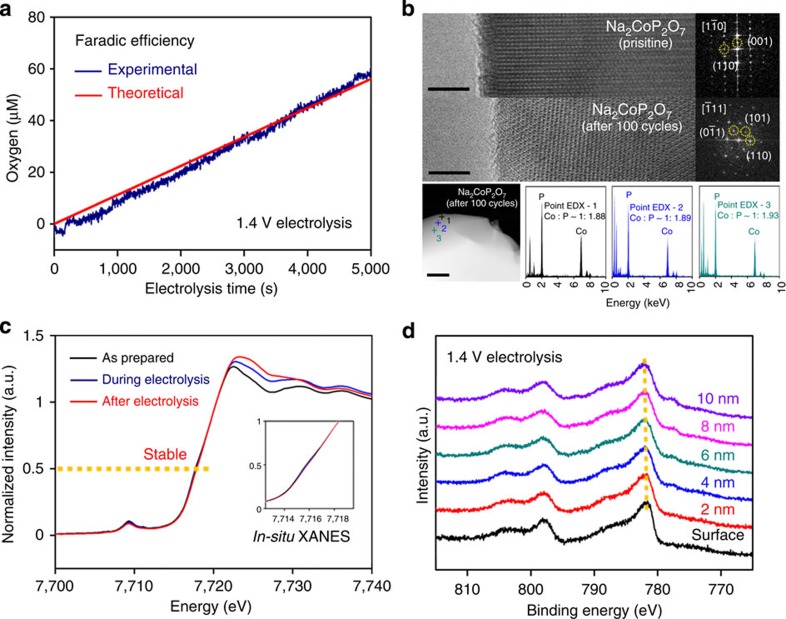
Faradaic efficiency and structural stability of Na_2_CoP_2_O_7_ during OER. (**a**) Faradic efficiency of Na_2_CoP_2_O_7_ measured by an oxygen sensor system. Bulk electrolysis was performed in a gas-tight electrochemical cell with the O_2_ sensor placed in the headspace. After initiating electrolysis at 1.4 V versus NHE, the percentage of O_2_ measured in the sensor on the FOXY probe was converted into the partial pressure of O_2_ in the headspace. The amount of evolved O_2_ molecules detected during bulk electrolysis and the theoretical amount of evolved O_2_ assuming a Faradaic efficiency of 100% are depicted by the blue line and red line, respectively. (**b**) HR-TEM images and FFTs at the surface region of Na_2_CoP_2_O_7_ before and after 100 continuous potential cycles from 0.7 to 1.5 V versus NHE (scale bar, 5 nm). STEM image of Na_2_CoP_2_O_7_ after potential cycles and point EDX spectrum from the surfaces to the inside region, showing similar Co/P ratios (scale bar, 20 nm). (**c**) *In-situ* XANES spectra corresponding to the Co K-edge of Na_2_CoP_2_O_7_ before, during and after bulk electrolysis at an applied potential of 1.5 V versus NHE for 2 h. The inset displays the enlarged XANES edge region. (**d**) XPS spectra of the Co 2*p* region of the surface of the bulk-electrolysed sample at an applied potential of 1.4 V versus NHE for 1 h.

The Faradaic efficiency of Na_2_CoP_2_O_7_ during OER was quantified using oxygen sensor system. About 60 μmol of O_2_ was produced during the electrolysis at 1.4 V versus NHE at a period of 5,000 s with a Faradaic efficiency of around ∼100%, indicating the measured current by CV is originated from the OER (Fig. 4a). With its proven ability for OER, facile mass production of Na_2_CoP_2_O_7_ powder using conventional synthesis methods and electrode-fabrication techniques enable us to produce large-scale oxygen evolution systems. The catalytic currents can be increased by integrating stacks of cells or by enlarging the size of the conducting substrate, and 180 mA of OER current can easily be acquired by stacking two cells with a geometric area of 64 cm^−2^ (Supplementary Fig. 7).

### Structural and chemical stability of Na_2_CoP_2_O_7_ during OER

To understand the relative stability of Na_2_CoP_2_O_7_ and NaCoPO_4_ compared with the other two compounds during OER cycles, XRD analysis was performed. The XRD patterns in Supplementary Fig. 8 reveal no appreciable peak changes after the electrolysis. For detecting the possible structural changes at the surface, we performed surface-sensitive HR-TEM and XPS analysis on the electrolysed samples. The HR-TEM image and Fast Fourier Transform of Na_2_CoP_2_O_7_ reveal the high crystallinity at the surface after 100 cycles (Fig. 4b). In addition, the NaCoPO_4_ exhibited high crystallinity, as shown in Supplementary Fig. 9. Point EDX analysis for bulk-electrolysed Na_2_CoP_2_O_7_ in Cs-corrected STEM mode showed no change in the Co/P ratio from the surface to the bulk region, further supporting their structural stabilities.

In contrast, an amorphous phase was formed on the surfaces of Li_2_CoP_2_O_7_ and LiCoPO_4_ after 100 potential cycles, whereas high crystallinity was found in pristine samples (Supplementary Fig. 10). This observation is in good agreement with a previous study on LiCoPO_4_, which gradually amorphized from the surface, resembling the local structure of Co-Pi (ref. 27). Moreover, the point EDX spectra for the bulk-electrolysed Li_2_CoP_2_O_7_ and LiCoPO_4_ showed that the Co/P ratio of the amorphized surface region is ∼2, which is well accordant to that of Co-Pi (refs 8, 27). In contrast, the bulk crystalline regions of Li_2_CoP_2_O_7_ and LiCoPO_4_ have Co/P ratios of 0.5 and 1, respectively, which were matched to their bulk compositional ratios. In addition, the CV curves for Li_2_CoP_2_O_7_, LiCoPO_4_ and Co-Pi showed similar redox features before the catalytic waves and onset-potential voltage (Supplementary Fig. 11). From the amorphous character and electrochemically similar behaviours of Li_2_CoP_2_O_7_, LiCoPO_4_ and Co-Pi, it can be deduced that the surface structure undergo phase transformation into Co-Pi or Co-Pi analogues. Previous reports showed similar phase transformation into Co-Pi-like structures in LiCoPO_4_ and Ba_*x*_Sr_1–*x*_Co_0.8_Fe_0.2_O_3–*δ*_ during OER tests^27^,^28^. The high crystallinity and stability both at the surface and in the bulk of Na_2_CoP_2_O_7_ and NaCoPO_4_ are quite inspiring considering that most cobalt-containing materials undergo a transformation into an amorphous Co-Pi-like phase from the surfaces^27^,^28^,^30^.

*In-situ* XANES and XPS analysis of Na_2_CoP_2_O_7_ were performed to probe any irreversible variation of the cobalt valence states during the OER using an *in-situ* electrochemical cell^20^,^50^. The Co K-edge spectra of Na_2_CoP_2_O_7_ were measured from 2 to 3 h after initiation of electrolysis at 1.5 V versus NHE. The Co K-edge spectra remained nearly constant during and after bulk electrolysis at 1.5 V versus NHE (Fig. 4c), indicating that no major change in the cobalt oxidation state occurred. In contrast to Na_2_CoP_2_O_7_, the Co K-edge spectra of the electrolysed Li_2_CoP_2_O_7_ and LiCoPO_4_ shifted towards the higher-energy region compared with that of the as-prepared sample, implying that cobalt oxidation occurred during the OER (Supplementary Fig. 12). Moreover, the Co 2p XPS peaks from the surface to a depth of 10 nm of the bulk-electrolysed Na_2_CoP_2_O_7_ and NaCoPO_4_ showed negligible variation (Fig. 4d and Supplementary Fig. 13). In contrast, the XPS depth profiling from the surface to 12-nm depths of cycled Li_2_CoP_2_O_7_ and LiCoPO_4_ showed that the surface Co 2*p* peaks shifted towards higher energy compared with those of the bulk, further supporting their structural change on electrolysis (Supplementary Fig. 13).

**Figure 5:**
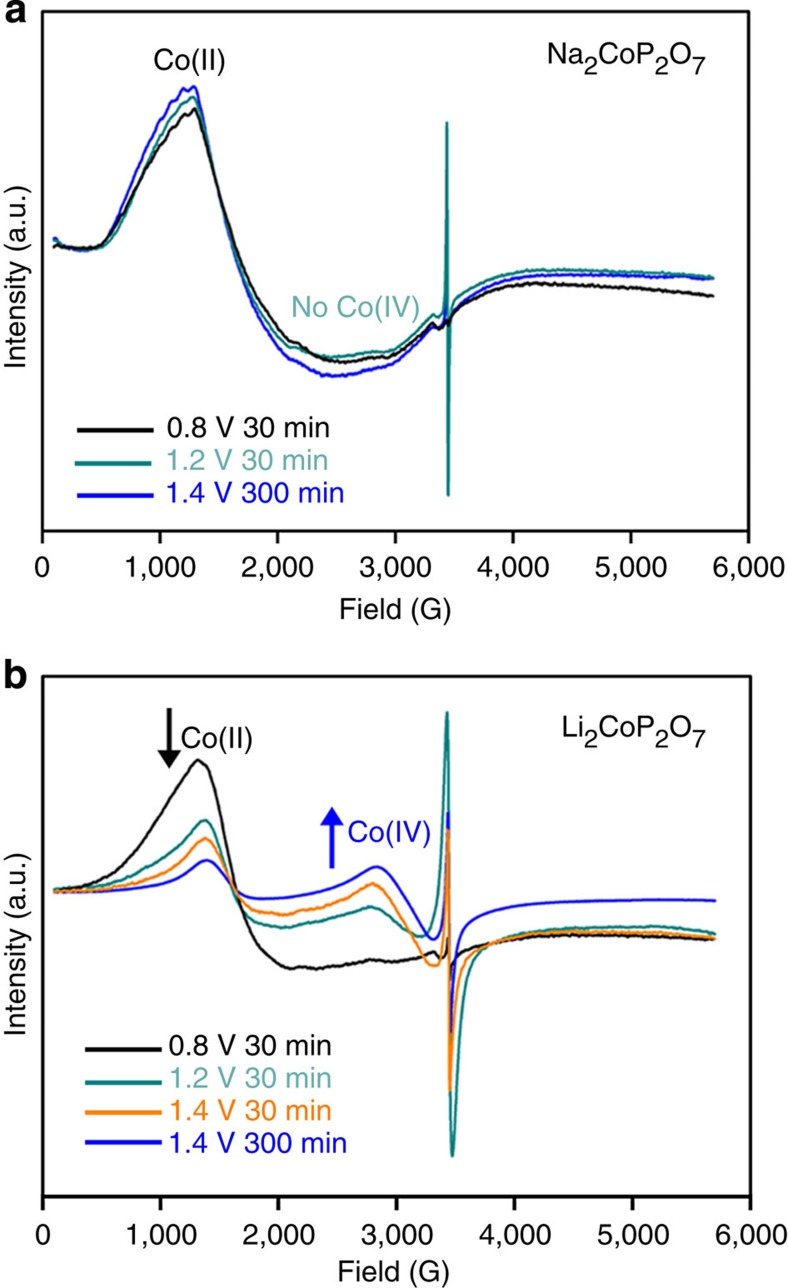
Continuous wave X-band EPR spectra for Na_2_CoP_2_O_7_ and Li_2_CoP_2_O_7_ on bulk electrolysis. (**a**) EPR spectra for Na_2_CoP_2_O_7_ at 0.8 V for 30 min (black), 1.2 V for 30 min (green) and 1.4 V for 300 min (blue). Only Co(II) signals at *g*_eff_≈5 appeared on bulk electrolysis. Here, the signal at *g*=2 is originated from carbon. (**b**) EPR spectra for Li_2_CoP_2_O_7_ at 0.8 V for 30 min (black), 1.2 V for 30 min (green), 1.4 V for 30 min (orange) and 1.4 V for 300 min (blue). Contrary to Na_2_CoP_2_O_7_, a decrease in Co(II) signals with a concurrent increase in Co(IV) signals (*g*_eff_≈2.3) was observed with the increase in applied voltage and time.

EPR analysis clearly showed the different structural stabilities of Na_2_CoP_2_O_7_, NaCoPO_4_, Li_2_CoP_2_O_7_ and LiCoPO_4_ during the OER tests by direct observations of Co(II) and Co(IV) species. The continuous-wave X-band EPR spectrum for Na_2_CoP_2_O_7_ only exhibited a characteristic *S*=3/2, Co(II) signal at *g*_eff_≈5 after bulk electrolysis at various potentials and times (Fig. 5a). Even 300 min of bulk electrolysis at 1.4 V versus NHE did not trigger a change in the Co EPR signal. Here, the signal at *g*=2 resulted from the carbon support (Supplementary Fig. 14). Invariance of the EPR signals on electrolysis supported the high structural stability of Na_2_CoP_2_O_7_.

In contrast, the distinct EPR signal around *g*_eff_≈2.3, which can be assigned to the low spin Co(IV) with its total spin *S*=1/2, appeared on bulk electrolysis of Li_2_CoP_2_O_7_ at an applied voltage of above 1.2 V versus NHE. With the decrease in the Co(II) signal, the Co(IV) signal increased when we raised the applied potential or electrolysis time, suggesting that significant oxidation of Co(II) into Co(IV) in Li_2_CoP_2_O_7_ occurred during the OER (Fig. 5b). Similar EPR trends were observed in previous reports using Co-Pi and they found that its catalytic resting state comprises Co(IV) species^30^,^51^. The increase in the Co(IV) signal with the concurrent decrease in Co(II) presented another evidence for structural change of Li_2_CoP_2_O_7_ into Co(IV)-containing Co-Pi-like structures during OER.

Similar with the case of Na_2_CoP_2_O_7_ and Li_2_CoP_2_O_7_, the EPR spectrum for bulk-electrolysed NaCoPO_4_ only showed a Co(II) signal at *g*_eff_≈5, whereas a distinct Co(IV) signal at *g*_eff_≈2.3 was found in bulk-electrolysed LiCoPO_4_ (Supplementary Fig. 15). In our studies the Co(III) signal did not appear, because the low-spin Co(III) with its total spin *S*=0 is EPR silent and high-spin Co(III) are rare and only existed in weak-field ligands, as described previously^51^. Although it is uncertain whether a small portion of Co(II) in Na_2_CoP_2_O_7_ or NaCoPO_4_ transformed into Co(III) during the OER, based on negligible change in the Co(II) signal on bulk electrolysis and no appearance of a Co(IV) signal, we believe that their dominant Co species during OER may be Co(II) or Co(II)/Co(III) mixed states. In addition, these EPR results supported phase transformation of Li_2_CoP_2_O_7_ and LiCoPO_4_ during the OER into Co(IV)-containing Co-Pi-like structures.

### Calculated surface structures of Na_2_CoP_2_O_7_

**Figure 6:**
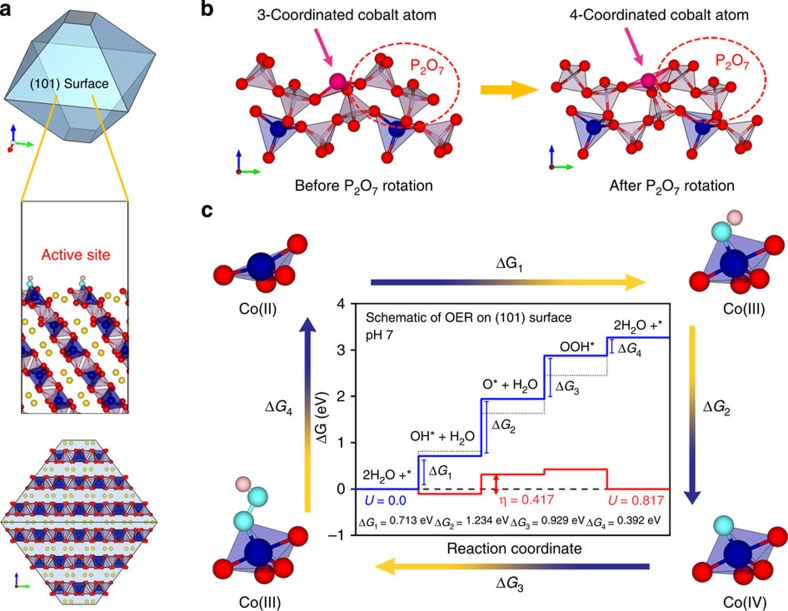
Density functional theory calculation for OER mechanism of Na_2_CoP_2_O_7_. (**a**) Wulff structure of Na_2_CoP_2_O_7_, showing the equilibrium crystal shape based on the relative surface energies and the enlarged (101) surface with active sites. (**b**) Local environment around the active cobalt atom located on the (101) surface before and after P_2_O_7_ rotation; the active cobalt atom is depicted in pink. (**c**) Schematic of the Gibbs free energy changes for the four elementary steps during the OER based on DFT calculations. The local structure and valence change of the cobalt atoms in the active sites are shown at the corners. The inset shows the free-energy landscape on the (101) surface of Na_2_CoP_2_O_7_ compared with an ideal catalyst at pH 7.0. An ideal catalyst, Na_2_CoP_2_O_7_ for *U*=0 and Na_2_CoP_2_O_7_ for *U*=0.817 are depicted by the dashed line, blue line and red line, respectively.

To understand the catalytic activity of Na_2_CoP_2_O_7_ with respect to its local structure on an atomistic level, we performed a series of first-principles calculations. The possible surface structures of Na_2_CoP_2_O_7_ were first calculated to estimate the equilibrium morphology at pH 7.0 (Supplementary Table 3). Based on the relative surface energies, the equilibrium crystal shape of Na_2_CoP_2_O_7_ was projected using Wulff constructions, which revealed that Na_2_CoP_2_O_7_ is mainly composed of a (101) surface with slight (001) surface exposure (Fig. 6a). It is worth noting that in the main (101) surface of Na_2_CoP_2_O_7_, the pyrophosphate ligands rotated around the cobalt atoms and generated a new Co–O bond, as illustrated in Fig. 6b. As shown in Supplementary Fig. 16, a general relaxation of surface atoms occurred and then new Co–O bonds were formed by rotation of the pyrophosphate ligands. After new Co–O bonds were formed, further relaxation occurred, creating the final surface structures of Na_2_CoP_2_O_7_ (101) plane. It should be noted that the pyrophosphate-group rotation in surface spontaneously occurs following an energetically favourable pathway with the decrease of Gibbs free energy. The structural flexibility of the P_2_O_7_ group could stabilize the under-coordinated transition metals on the surface^40^. As a result of the passivation by the pyrophosphate group, three-coordinated surface cobalt atoms initially formed after surface-cleaving step irreversibly transformed into a highly distorted tetrahedral geometry similar to the CoO_5_ TBP structure with a deficiency of one oxygen.

### OER catalytic mechanism of Na_2_CoP_2_O_7_

Given the well-established surface structures, we attempted to estimate the overpotential by sequentially calculating the Gibbs free energies for the four elementary steps occurring on the surface during the OER based on acid–base mechanism^18^,^22^,^31^. These four steps are used for modelling the thermochemistry of the OER^18^,^22^,^31^ and are described as follows:


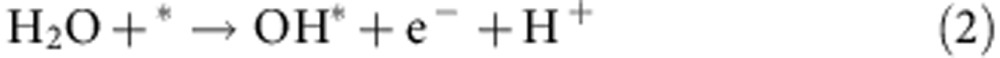



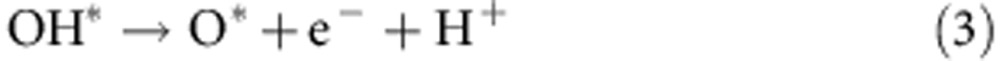



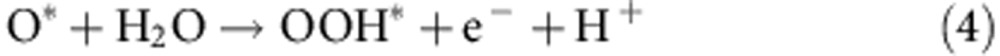



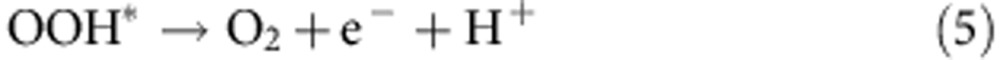


(*represents the active surface site where OH and O species can adsorb).

During the process, water molecules first adsorb on the active site on the surface and dissociate into a proton and OH* (step 1), which again splits into a proton and O* (step 2). The oxygen atom recombines with a water molecule to form an OOH* bond (step 3) and finally evolves as O_2_ gas (step 4). Our calculation model has limitations in neglecting the influence of the electric field on the double layer and the possible differences resulting from the types of proton donor (H_3_O^+^ or H_2_O). According to previous reports^18^,^52^,^53^, with this assumption, the Gibbs free energy of each step during the OER can be calculated as identical, regardless of the pH value. In this regard, we simply calculated the Gibbs free energy of each step in Na_2_CoP_2_O_7_ using the four-step model in acidic conditions.

Moreover, it should be noted that an alternative OER mechanism exists, the so-called direct coupling mechanism^54^,^55^. Recently, Wang and Van Voorhis^54^ revealed the new OER mechanism in cobalt oxide, demonstrating direct-coupling O–O bond formation can occur with a very low activation barrier, and steps other than O–O bond formation act as the RDS in cobalt oxides. In this stage, based on the relatively larger inter-cobalt distance in Na_2_CoP_2_O_7_ (5.15 Å) compared with that in cobalt oxide (∼2.85 Å) revealed by *in-situ* XAS analysis^26^, we focused on the acid–base mechanism for exploring the OER pathway in Na_2_CoP_2_O_7_.

During these four steps, the cobalt ion is sequentially oxidized from 2 to 3 (step 1) and 3 to 4 (step 2), and is reduced from 4 to 3 (step 3) and 3 to 2 (step 4), indicating the active participation of the cobalt redox reaction at every step of the OER. The detailed energy changes during the four elementary steps are shown for the two dominant surfaces, (101) and (001) surfaces, in Fig. 6c and Supplementary Fig. 17, respectively. Here, the dotted line represents the calculated Gibbs free energies at ideal charge transfer steps and the blue line represents those of Na_2_CoP_2_O_7_ when *U*=0, both at pH 7.0. The red line represents the net Gibbs free energy changes of Na_2_CoP_2_O_7_ when *U*=0.817, which represents the additionally required energy for the reaction to proceed (equivalent to the overpotential) in each step during the OER. Probing the Gibbs free energies for these steps reveals that the (101) surface exhibits a significantly lower overpotential of 0.417 eV than that of the (001) surface of 1.117 eV (Fig. 6c and Supplementary Fig. 17). It is noteworthy that the magnified CV curves in Supplementary Fig. 4 show that the current increased dramatically near the overpotential of 0.42 V, supporting the validity of our calculations, although experimental overpotential may slightly vary with the current density.

The individual steps of the water oxidation are closely investigated in the following. As a first step, we monitored the Gibbs free energy change for OH* formation (step 1) on the (101) surface in our system. As the absorption of water molecules on the surface is the initial step of the OER, it is generally perceived that the absorption energy of water molecules highly affects the OER activity^52^,^53^,^54^,^55^,^56^,^57^,^58^,^59^,^60^. Norskov and colleagues^52^,^53^,^58^ reported the importance of the adsorption energies of adsorbents, such as *OH and *OOH species, on the OER activities on metal-oxide surfaces and a universal scaling relation between *OH and *OOH adsorption energy was found. Recently, Liu *et al*.^59^ and Kim *et al*.^60^ showed that oxygen or cobalt vacancies with high binding energies with water molecules lead to a significant enhancement of OER catalytic capabilities. Interestingly, the Gibbs free energy for step 1 in our system only requires 0.713 eV, which is lower than the energy needed (0.817 eV) to initiate water oxidation ideally at pH 7.0, implying a facile water adsorption on surface cobalt atoms in the (101) plane. This facile adsorption originates from the unique structural characteristic of the reorganized surface cobalt atoms; pyrophosphate ligands induced highly distorted *T*_d_ coordination, which has an open site, where oxygen adsorbants can easily bind. Indeed, previous reports showed that similar distorted metal geometries with open coordination sites have an advantage in the uptake of oxygen adsorbants^59^,^60^,^61^,^62^. This finding strongly suggests that the distorted local cobalt geometry induced by the reorganization of pyrophosphate ligands contributed to a favourable water adsorption and further water oxidation catalysis.

Compared with facile OH* formation, O* formation (step 2) requires the highest barrier (0.417 eV) among the four steps, indicating that this step is the rate-limiting process of the water oxidation catalysis of Na_2_CoP_2_O_7_. This theoretical value is comparable in magnitude to that of the active cobalt-oxide β-CoOOH (0.48 eV), which was selected as a representative for amorphous Co-Pi (ref. 31). Considering that RDS in conventional cobalt oxides including β-CoOOH is generally known as OOH* formation^30^,^31^, the limiting step of O* formation in Na_2_CoP_2_O_7_ is worthwhile to be noticed. However, it should be noted that steps other than O–O bond formation can be the RDS by direct O–O coupling^54^. We speculate that the highest barrier residing in step 2 of Na_2_CoP_2_O_7_ is related to the inherent nature of the high cobalt oxidation potential in the phosphate framework^42^,^43^,^45^. Much higher redox potentials for cobalt (Co(II)/Co(III) or Co(III)/Co(IV)) are generally observed in polyanion frameworks such as phosphates and pyrophosphates compared with oxides because of the inductive effect^42^,^43^,^45^. As step 2 requires the oxidation of the Co(III) to Co(IV) in pyrophosphates, a substantially higher energy will be required to oxidize the Co(III) ion than in oxides^42^,^43^,^45^. It is noteworthy that the redox potentials of cobalt ions in oxides typically shift up by ∼1 V in phosphates^42^,^45^. The higher energy for oxidizing Co(III) in Na_2_CoP_2_O_7_ may make O* formation the RDS.

Another interesting characteristic of Na_2_CoP_2_O_7_ was found in the adaptable local cobalt structure during the OER (corners of Fig. 6c). Although the OER proceeds in five- and six-coordinated polyhedron in conventional cobalt oxides^26^,^30^,^31^, Na_2_CoP_2_O_7_ used four- and five-coordination geometries accompanying the change of cobalt valence. Specifically, with the valence change from 2 to 3 in step 1, the oxygen coordination number of cobalt increased from 4 to 5 by adsorbing the OH species. Then, further oxidation to Co(IV) occurs by detaching the proton from the adsorbed OH and the remaining oxygen bound to Co(IV) recombines with a water molecule to make OOH, reducing the cobalt to III. During these two redox steps (step 2 and 3), the five-coordination geometry was retained without a significant change in the atom positions. As under-coordinated transition metals are generally unstable and are prone to induce surface reconstruction^63^,^64^, the stable performance of the four/five coordination of cobalt is remarkable. This performance was possible mainly because of the rotation of the flexible pyrophosphate group, which readily forms additional Co–O bonds at the surface, indicating the important interplay between the cobalt ion and phosphate polyanion.

We performed additional computations to explore the theoretical OER mechanism for NaCoPO_4_ and β-CoOOH (Supplementary Fig. 18). β-CoOOH was introduced for representing Co-Pi-like structures at the surface of Li_2_CoP_2_O_7_ and LiCoPO_4_ during the OER. The four OER steps of the dominant NaCoPO_4_ surface, the (010) plane, showed that theoretical overpotential of NaCoPO_4_ was 0.60 V with RDS of O* formation. In case of β-CoOOH, the 
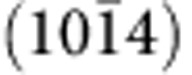
 plane was selected as the most catalytically active surface, on the basis of a previous study based on the acid–base mechanism. The theoretical overpotential of β-CoOOH was calculated as 0.44 V with RDS of *OOH formation, which was in line with a previous report^31^.

Finally, we compared the similar and different characteristics of Na_2_CoP_2_O_7_ and Co-Pi (β-CoOOH) in Table 1. Whereas these two types of catalyst exhibited similar electrochemical performances, their underlying catalytic mechanisms showed strikingly different properties. In terms of structure, Na_2_CoP_2_O_7_ has four/five-coordination geometry inside the crystalline structure during OER, whereas Co-Pi has *O*_h_-based five/six coordination in an amorphous structure^8^,^30^,^31^. The dominant Co species of Na_2_CoP_2_O_7_ during OER is Co(II) or Co(II)/Co(III), whereas that of Co-Pi is known as Co(III)/Co(IV)^26^,^30^. Finally, the theoretically calculated RDE of Na_2_CoP_2_O_7_ is *O formation, whereas that of Co-Pi is *OOH formation (acid–base mechanism)^30^,^31^. Based on these comprehensive comparisons, we think that Na_2_CoP_2_O_7_ can be a good model system to explore new OER chemistry beyond conventional *O*_h_-based cobalt-oxide materials.

## Discussion

In summary, we explored the abilities of four phosphate/pyrophosphate-based, well-known cobalt redox-active materials as water oxidation catalysts and found a highly active and stable material, Na_2_CoP_2_O_7_. Whereas previously reported cobalt-containing materials underwent significant phase transformation during the OER, Na_2_CoP_2_O_7_ exhibited excellent catalytic activity and phase stability. Moreover, mechanistic studies for the OER of Na_2_CoP_2_O_7_ were performed by varying the pH and phosphate buffer strength of the electrolyte. Density functional theory calculations revealed that Na_2_CoP_2_O_7_ have lower theoretical overpotential (∼0.42 eV) compared with the most active cobalt-oxide phase, CoOOH (∼0.48 eV), theoretically verifying its high catalytic activity. In addition, we found the possible OER pathway in Na_2_CoP_2_O_7_ that used four- and five-coordinated cobalt atoms stabilized by actively rotating pyrophosphate groups. Through the experimental and theoretical comparison, we found that the underlying catalytic mechanism of Na_2_CoP_2_O_7_ was notably different to that of the conventional cobalt oxides, in terms of RDS, local cobalt structure and cobalt valence state during the OER. We believe that using polyanion-based cobalt compounds can be an interesting new direction for the development of new efficient and stable catalysts, and the discovery of new catalytic pathways in various transition metal geometries. We expect that this result can broaden the current oxide-based picture of the water oxidation complex and give hints at a possible effect of the polyanion on OER catalytic abilities.

## Methods

### Material synthesis

Na_2_CoP_2_O_7_ and NaCoPO_4_ powders were synthesized using the conventional solid-state method. Stoichiometric amounts of Na_2_CO_3_ (ACS reagent, ⩾99%, Aldrich), CoC_2_O_4_ (⩾99%, Kojundo) and (NH_4_)_2_HPO_4_ (ACS reagent 98%, Aldrich) were wet-ball milled in acetone media and heated at 300 °C under Ar media for 6 h. After grinding, the pelletized samples were calcined at 600 °C and 750 °C under Ar media for 6 h for Na_2_CoP_2_O_7_ and NaCoPO_4_, respectively. Li_2_CoP_2_O_7_ and LiCoPO_4_ powders were synthesized using the stoichiometric amounts of Li_2_CO_3_ (ACS reagent, ⩾99%, Aldrich), CoC_2_O_4_ (⩾99%, Kojundo) and (NH_4_)_2_HPO_4_ (ACS reagent 98%, Aldrich). The precursors were mixed by ball milling, heated at 300 °C under Ar media for 6 h, pelletized and calcined at 600 °C for Li_2_CoP_2_O_7_ and 800 °C for LiCoPO_4_ under Ar media for 6 h. Amorphous Co-Pi was synthesized according to the previous report^8^.

### Material characterization

Powder XRD patterns were measured using a Bruker D-8 Advance X-ray diffractometer using Cu Kα radiation (*λ*=1.54056 Å) from 10° to 70° with a step size of 0.01°. Structural analysis of materials was performed by Rietveld refinement method using FullProf software. TEM images were obtained using a field-emission TEM (Philips Tecnai F20) operating at 200 kV. HR-TEM images and FFTs of the materials were used to analyse surface morphology. Point EDX analysis was performed under the Cs-corrected STEM mode and the spectra from three different regions were acquired with a Quanta 3D FEG (FEI, The Netherlands). The XAS experiments were performed at beamline 8C at the Pohang Accelerator Laboratory, Republic of Korea. The oxidation state corresponding to the Co K-edge in each sample was investigated using XANES analysis. *In-situ* XANES spectra were collected in fluorescence mode with an electron energy of 2.5 GeV and stored in the current of 250 mA top-up mode. XPS spectra were performed to examine the oxidation state of the Co 2*p* peaks from surface to depth of 10 nm of the catalyst after bulk electrolysis. XPS analysis was performed five times at an increment of 2 nm etching from the surface. The data were attained with passing energy 23.5 eV with a step size of 0.05 eV by electron spectroscopy (Sigma Probe, Thermo VG Scientific, UK). Brunauer–Emmett–Teller analysis was performed to analyse the surface area of the catalyst. The sample was loaded in the Brunauer–Emmett–Teller analyser (Tristar II 3020 Micromeritics, USA) under an N_2_ adsorption environment.

### Electrochemistry

The electrochemical measurements were performed in a three-electrode system using a potentiostat (CHI 760C, CH Instruments, Inc.). Pt foil (2 cm × 2 cm × 0.1 mm, 99.997% purity, Alfa Aesar) was used as the counter electrode and Ag/AgCl electrode (BASi, 3 M NaCl) was used as a reference electrode. The working electrode was prepared by a previously reported spin-coating method^21^,^23^. The catalyst with neutralized Nafion solution was dispersed in purified deionized water using sonication and was spin-coated onto an fluorine-doped tin oxide substrate. After the electrode was dried at 80 °C for 5 min, the loaded catalyst weight was carefully measured using a micro weighing electronic scale (Sartorius Micro Balance). A pH 7.0 sodium phosphate buffer with buffer strength 0.5 M was used as the electrolyte, and the electrolyte was degassed with high-purity N_2_ before every electrochemical measurement. The OER curves were corrected using the averaged value of the forward and reverse CV currents for polarization compensation and further iR-compensation using *V*=*V*_applied_−iR was performed. After polarization and iR compensation, each OER value was normalized to the total surface area of the catalysts on the substrate. The electrochemical potentials were converted to the NHE scale by following equation: *E*(NHE)=*E*(Ag/AgCl)+0.197 V.

To prepare electrolytes with different pH values from 5.0 to 8.8, 0.5 M phosphate buffer was used with varying ratios of NaH_2_PO_4_ and Na_2_HPO_4_. Over pH 9, the pH was adjusted by addition of NaOH into the phosphate buffer (pH 8.8) in the customary manner. To determine the effect of the phosphate buffer concentration on the activity, different phosphate buffer concentrations from 0.1 to 1 M were prepared and different amounts of NaClO_4_ were added to maintain the same solution conductivity regardless of the phosphate buffer concentrations.

### EPR measurement

X-band continuous-wave EPR spectra were obtained using a Bruker EMX/Plus spectrometer equipped with a dual-mode cavity (ER 4116DM). A liquid He quartz cryostat (Oxford Instruments ESR 900) combined with gas flow controller (Oxford Instruments ITC 503) was introduced to control the low temperatures (∼5.7 K). For EPR measurement, 15 mg of Na_2_CoP_2_O_7_, NaCoPO_4_, Li_2_CoP_2_O_7_ and LiCoPO_4_ catalyst electrodes were prepared as carbon-paste type. After bulk electrolysis at the specific voltages and times, the electrodes were removed from electrolyte, followed by quick drying using an Ar (99.999 %) gun. Then, the solid-state catalysts were carefully collected by a razor blade and were transferred to an EPR tube under Ar (99.999 %) atmosphere. The EPR tube was immediately frozen and stored at 77 K using liquid nitrogen before EPR measurement. The elapsed time from the end of electrolysis to freezing of the EPR tube was ∼5 min. The following parameters were used: microwave frequency=9.64 GHz, modulation amplitude=10 G, modulation frequency=100 kHz, microwave power=0.94 mW and temperature=5.7 K. Ten scans were added for obtaining each spectrum.

### First-principles calculations

All the free energies in this work were calculated using the generalized gradient approximation (GGA) and the Perdew–Burke–Ernzerhof functional for the exchange correlation to the density functional theory^65^. The projected augmented wave method was used, as implemented in the Vienna *ab initio* simulation package^66^. The Hubbard *U* parameter (GGA+*U*) with *U*=3.5 eV was used to accurately calculate the electron correlation within the *d* states in a cobalt ion^67^,^68^,^69^,^70^. A 3D slab model with periodic boundary conditions was used with an energy cutoff of 500 eV and an appropriate *γ*-point, only *k*-points mesh, was selected to ensure that the total energies converged within 5 meV per formula unit of Na_2_CoP_2_O_7_.

### Wulff construction and surface properties

To calculate the surface energies and Wulff shape of Na_2_CoP_2_O_7_, the initial unrelaxed surface structures were carved out from the fully relaxed bulk structure of Na_2_CoP_2_O_7_ with P42mnm symmetry, which is a less distorted structure with higher symmetry than the experimental P21cn structure. The selection of surfaces to investigate was limited to low-index surfaces and guided by favouring surfaces with preservation of the PO_4_ tetrahedron, which led to the following set of surfaces: (100), (001), (110), (210), (101), (111) and 
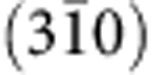
.

All the surface structures were calculated within the GGA+U scheme as defined above, using a supercell with atomic layers over 15 Å and vacuum slabs over 20 Å along the *z* direction using a convergence test to obtain the adsorption energies and to recover the bulk magnetic ordering of the centre layers (convergence within 1 meV Å^−2^ for the surface energies). The surface energies were calculated from equation (6) on either stoichiometric or non-stoichiometric slabs for which the top- and bottom-most cobalt pyrophosphate layers were relaxed below the force threshold of 0.05 eV Å^−1^.


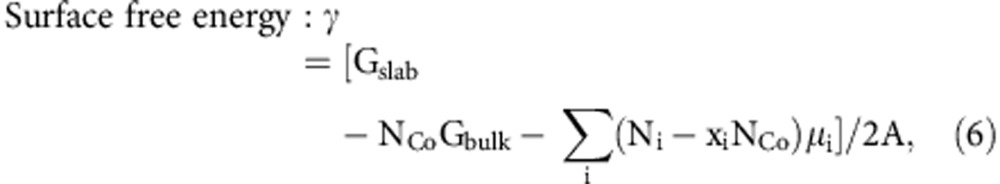


with surface area 2A, free energy *G*_slab_, chemical potentials *μ*_i_, the number of excess adsorbed species over bulk *N*_*i*_−*x*_*i*_*N*_Co_ (where *x*_*i*_ is the number of atoms per bulk formula).

The chemical potentials *μ*_i_ of the adsorbents were taken relative to the free energy of liquid water and hydrogen gas, written explicitly as functions of pH and applied potential U:


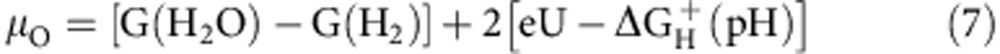



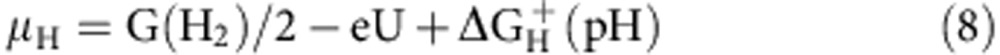



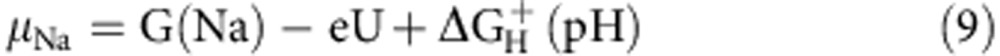


where U is the potential measured against NHE at standard conditions and Δ*G*_H_^+^(pH) is −*k*_B_*T* log(pH).

### Gibbs free energy changes in water oxidation steps

We defined the OER steps similar to equations (2, 3, 4, 5) in the manuscript. The Gibbs free energy changes for the water oxidation steps (equations (2, 3, 4, 5) in the manuscript) were calculated using the following equations:


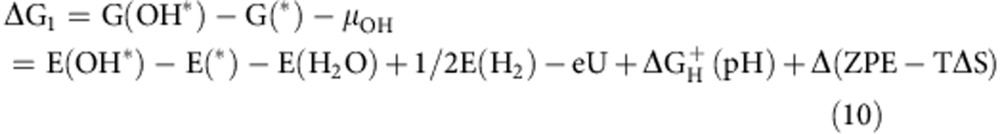



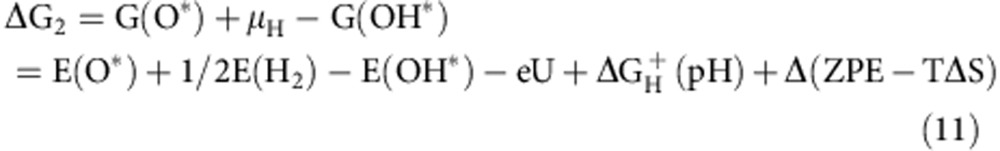



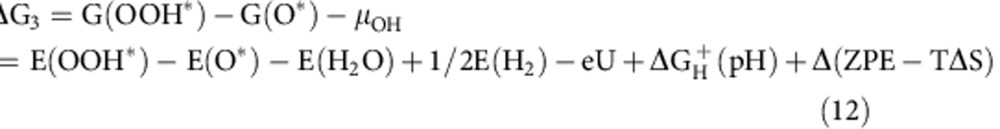



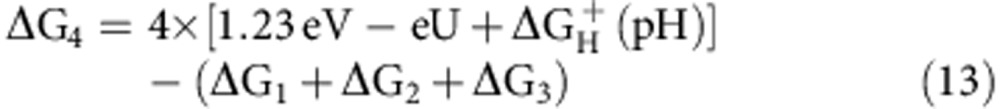


where the density functional theory energies, zero point energy and entropy correction values are listed in the Supplementary Table 4.

## Additional information

**How to cite this article:** Kim, H. *et al*. Coordination tuning of cobalt phosphates towards efficient water oxidation catalyst. *Nat. Commun.* 6:8253 doi: 10.1038/ncomms9253 (2015).

## Supplementary Material

Supplementary Information Supplementary Figures 1-18, Supplementary Tables 1-4 and Supplementary References

## Figures and Tables

**Table 1:** Comparison between Na_2_CoP_2_O_7_ and CoO_x_.

**Catalyst**	**Na**_**2**_**CoP**_**2**_**O**_**7**_	**CoOx (Co-Pi,** ***β*****-CoOOH)**	**Refs**
*Electrochemical performance*			
Catalyst type	Powder (crystalline)	Electrodeposited thin film (amorphous)	8
Experimental overpotential value	560 mV (1 mA cm^−2^)	410 mV (1 mA cm^−2^)	8
pH (electrolyte composition )	pH 7 (0.5 M phosphate)	pH 7 (0.1 M phosphate)	8
Tafel slope	Approximately 80∼90 mV dec^−1^	60 mV dec^−1^	8,30
pH dependence	67 mV	64 mV	47
Buffer concentration dependence	Zeroth order dependence	Zeroth order dependence	47
			
*Structural/chemical property during OER*			
EPR	2+ or 2+/3+ mixture	3+/4+ mixture	30
*In-situ* XANES	2+	⩾3+	26
HR-TEM at surface (EDX)	High crystallinity (Co: *P*=1:2, TEM)	Amorphous phase (Co: *P*=2∼3:1, SEM)	8
			
*OER mechanism*			
Cobalt coordination (Pristine state)	4 (Tetrahedral)	6 (Octahedral)	26,30
Cobalt coordination (During OER)	4↔5	5↔6	31
OER active surface	(101)	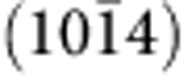	31
Rate determining Step	OH*→O*	O*→OOH* (acid–base mechanism)	31
Theoretical overpotential value	417 mV	480 mV (ref) 437 mV (our work)	31

EDX, scanning transmission electron microscopy; EPR, electron paramagnetic resonance; HR-TEM, high-resolution transmission electron microscopy; OER, oxygen evolution reaction; XANES, X-ray absorption near edge spectroscopy.
