# Supplementary material for: Coordination tuning of cobalt phosphates towards efficient water oxidation catalyst
Source: Nat Commun. 2015 Sep 14;6:8253. doi: 10.1038/ncomms9253 (PMC4579784; doi:10.1038/ncomms9253)
Supplement: Supplementary Information — Supplementary Figures 1-18, Supplementary Tables 1-4 and Supplementary References [file ncomms9253-s1.pdf]

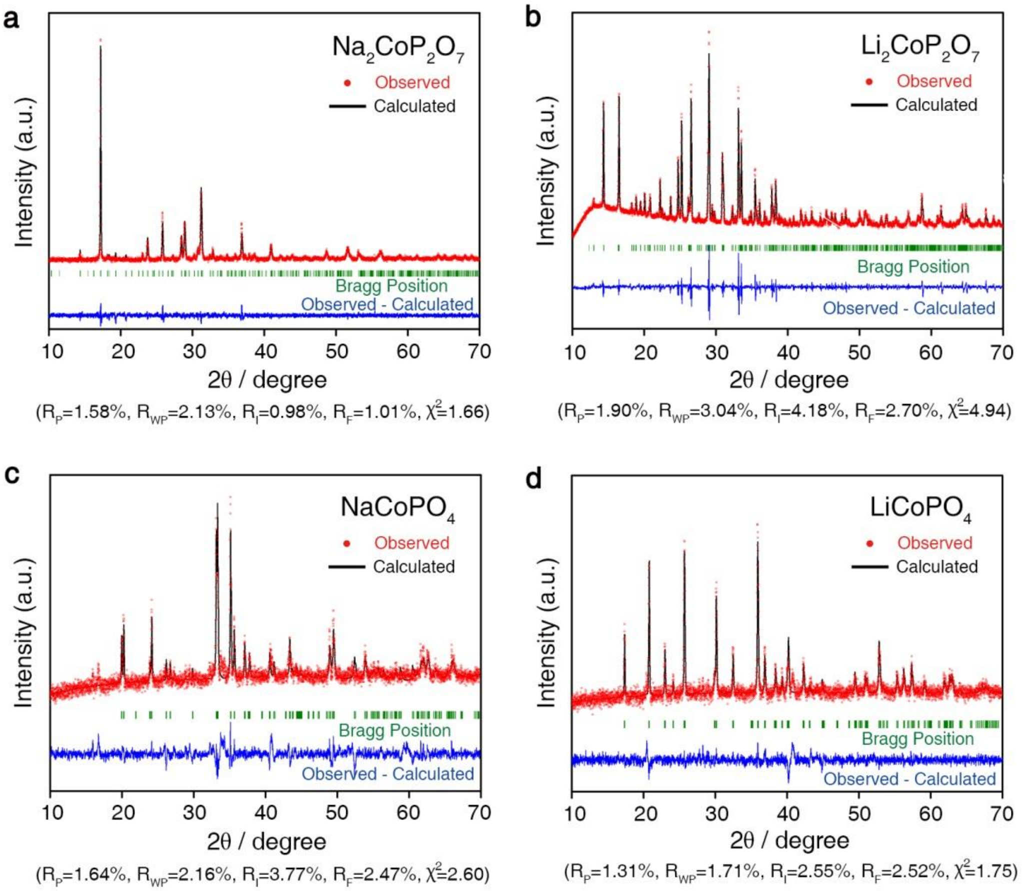

Supplementary Figure 1. Structural information of the cobalt phosphate catalysts. Rietveld refinement patterns of high-resolution X-ray diffraction data of the (**a**) $Na_2CoP_2O_7$, (**b**) $Li_2CoP_2O_7$, (**c**) $NaCoPO_4$, and (**d**) $LiCoPO_4$. Red dots (experimental data points), black line (calculated powder pattern), green ticks (Bragg positions) and blue line (difference between the observed and calculated patterns).

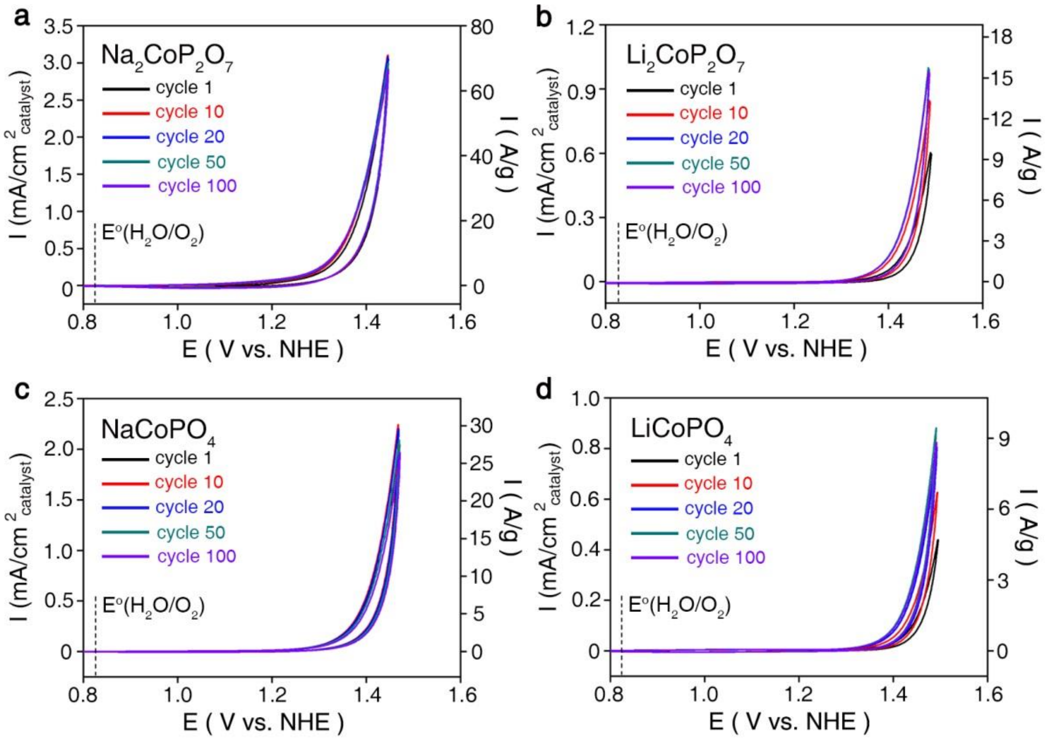

Supplementary Figure 2. Cyclic voltammetry curves for the cobalt phosphate catalysts. Curves of (**a**) $Na_2CoP_2O_7$, (**b**) $Li_2CoP_2O_7$, (**c**) $NaCoPO_4$, and (**d**) $LiCoPO_4$ during 100 times of voltammetry cycling in 0.5 M sodium phosphate buffer at neutral condition. The current value was normalized to the surface area (left) and total weight (right) of the catalyst. The thermodynamic potential for water oxidation is marked at 0.816 V vs. NHE (pH 7.0).

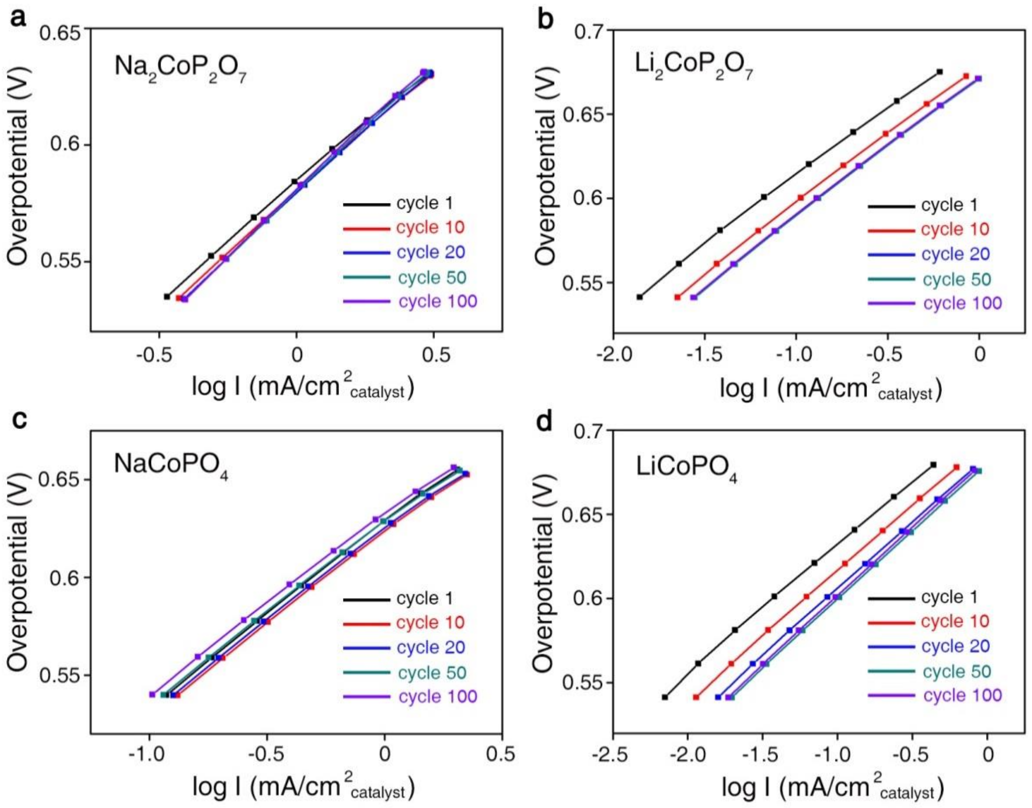

Supplementary Figure 3. Tafel analysis for the cobalt phosphate catalysts. Tafel plots of (**a**) $Na_2CoP_2O_7$, (**b**) $Li_2CoP_2O_7$, (**c**) $NaCoPO_4$, and (**d**) $LiCoPO_4$ during 100 times of voltammetry cycling. The current value was normalized by the total surface area of the catalyst.

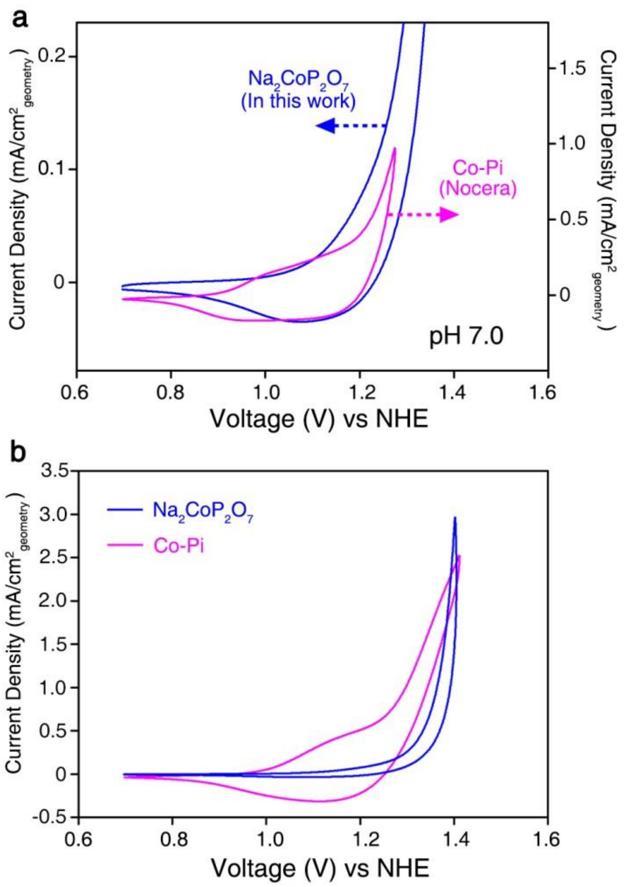

Supplementary Figure 4. Comparative cyclic voltammetry curves for $Na_2CoP_2O_7$ and Co-Pi. Comparison of cyclic voltammetry curves scans for $Na_2CoP_2O_7$ and electrodeposited Co−Pi from (**a**) 0.7 V to 1.3 V vs. NHE and (**b**) 0.7 V to 1.5 V vs. NHE at a scan rate of 10 mV s^{-1}. The current value was normalized to the area of the electrode. For the Co−Pi films, the loadings were 1000 mC cm^{-2} in 0.1 M potassium phosphate buffer at pH 7.0.

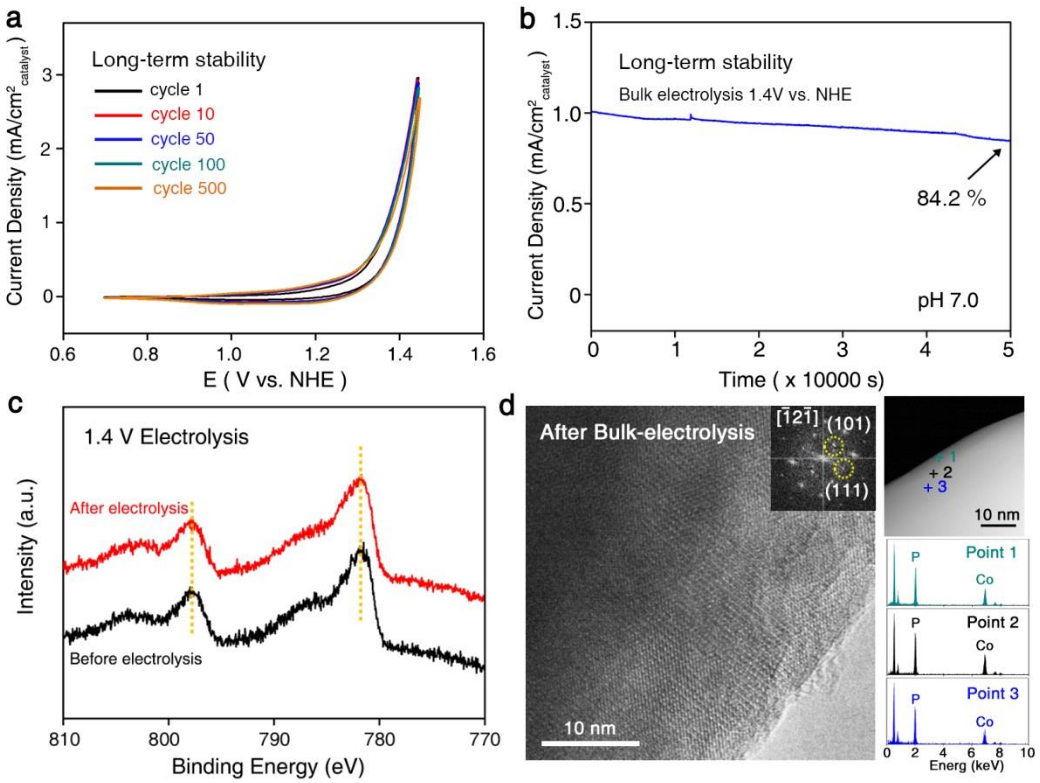

Supplementary Figure 5. Long-term stability tests for $Na_2CoP_2O_7$. (**a**) Cyclic voltammetry curves for 500 times of voltammetry cycling in 0.5 M sodium phosphate buffer at neutral condition. (**b**) Bulk-electrolysis was performed at a constant potential of 1.4 V vs. NHE at pH 7.0 for 50,000 s. (**c**) XPS spectra of the Co 2p region of the surface before and after bulk-electrolysis (**d**) HR-TEM, FFT image, and point STEM-EDX at the surface region of $Na_2CoP_2O_7$ after the bulk-electrolysis.

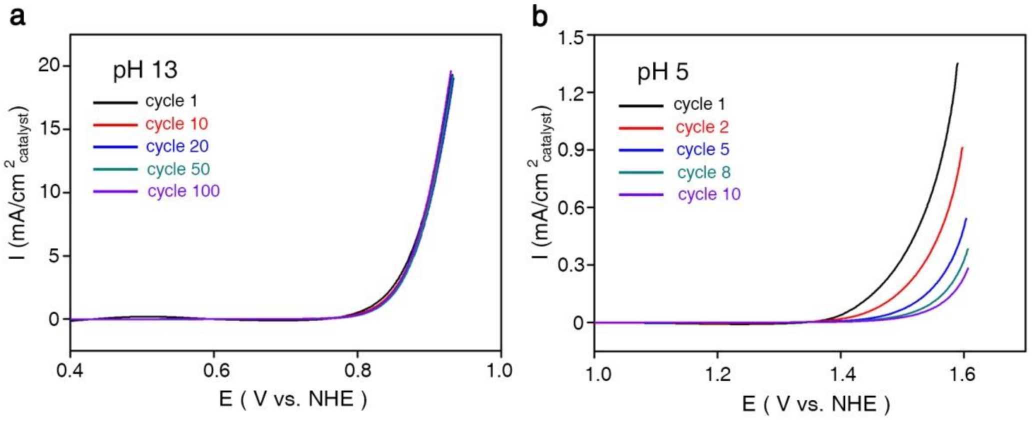

Supplementary Figure 6. Stability tests for $Na_2CoP_2O_7$ under basic and acidic conditions. Cyclic voltammetry curves of $Na_2CoP_2O_7$ for 100 times of voltammetry cycling in basic condition at pH 13 (**a**) and 10 voltammetry cycles under acidic condition at pH 5 **(b**).

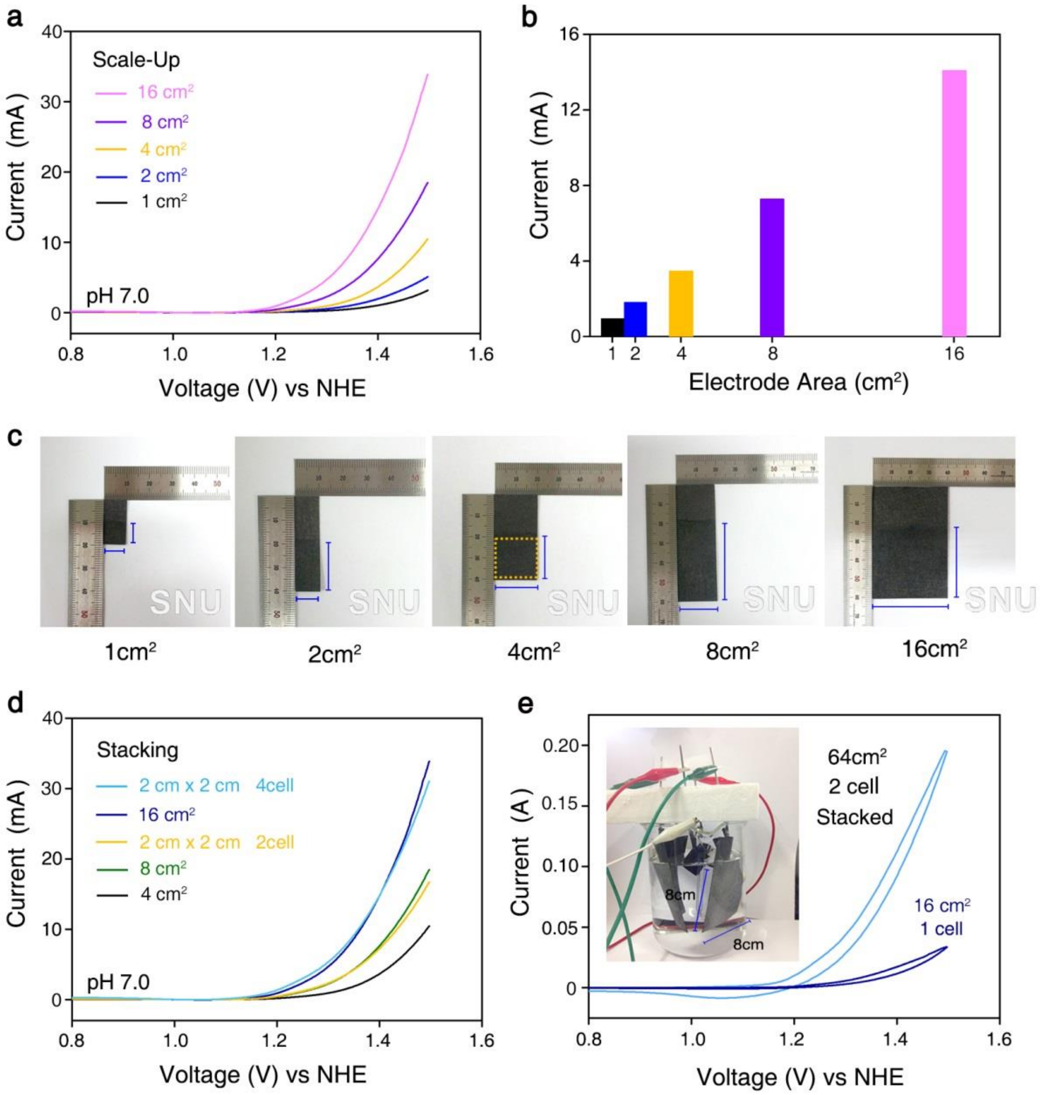

Supplementary Figure 7. Large-scale oxygen-evolution systems using scale-up and stacking process with $Na_2CoP_2O_7$ catalyst. (**a**) Cyclic voltammetry curves using various sizes of electrode. (**b**) The OER current at an applied voltage of 1.4 V vs. NHE was linearly enhanced as the electrode area increased. (**c**) Optical image of various-sized electrodes. (**d**) Cyclic voltammetry curves formed by integrating stack of cells. (**e**) Cyclic voltammetry curves of stacking two cells with an electrode area of 64 cm^2. Inset shows the optical image of stacked cells.

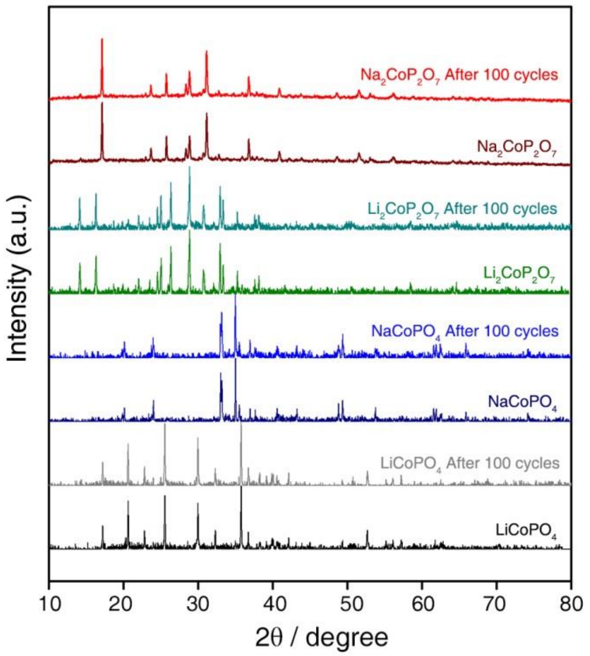

Supplementary Figure 8. XRD patterns of the cobalt phosphate catalysts before and after OER. XRD patterns of (a) $Na_2CoP_2O_7$, (b) $Li_2CoP_2O_7$, (c) $NaCoPO_4$, and (d) $LiCoPO_4$ before and after 100 continuous potential cycling from 0.7 V to 1.5 V vs. NHE. All the cyclic voltammetry analyses were performed in 0.5 M sodium phosphate buffer (pH 7.0) at a scan rate of 10 mV sec^{-1}.

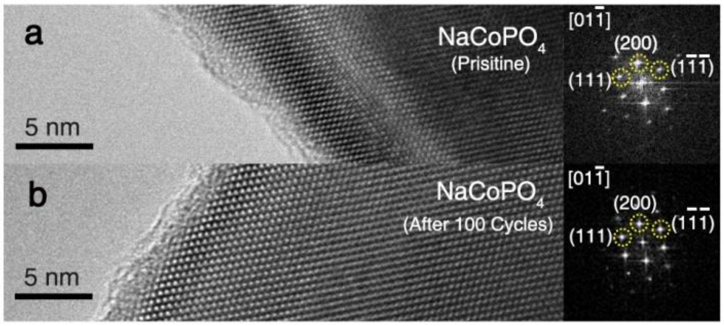

Supplementary Figure 9. HR-TEM analysis of $NaCoPO_4$. HR-TEM images and FFTs of the surface regions of (**a**) $NaCoPO_4$ in pristine condition, and (**b**) $NaCoPO_4$ after 100 continuous potential cycles from 0.7 V to 1.5 V vs. NHE. All potential cycling analyses were performed in 0.5 M sodium phosphate buffer (pH 7.0) at a scan rate of 10 mV sec^{-1}.

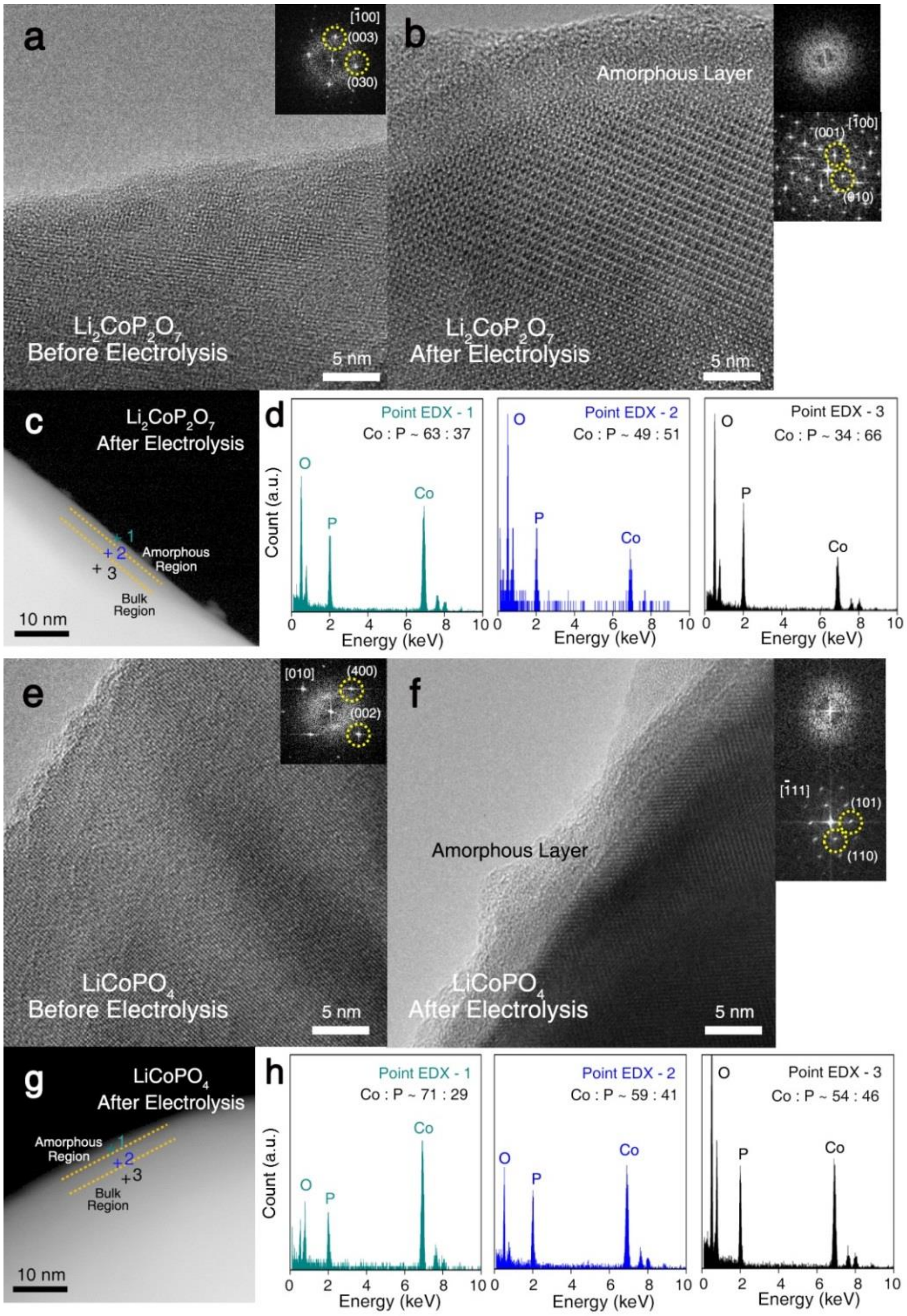

Supplementary Figure 10. HR-TEM and EDX analysis of $Li_2CoP_2O_7$ and $LiCoPO_4$. HR-TEM images and FFTs of the surface regions of $Li_2CoP_2O_7$ before (**a**) and after (**b**) electrolysis. (**c**) STEM image and (**d**) point EDX spectrum from the surfaces to the inside region. HR-TEM images and FFTs of $LiCoPO_4$ before (**e**) and after (**f**) electrolysis at the surface regions. (**g**) STEM image and (**h**) point-EDX spectrum showing the Co/P ratio at each region.

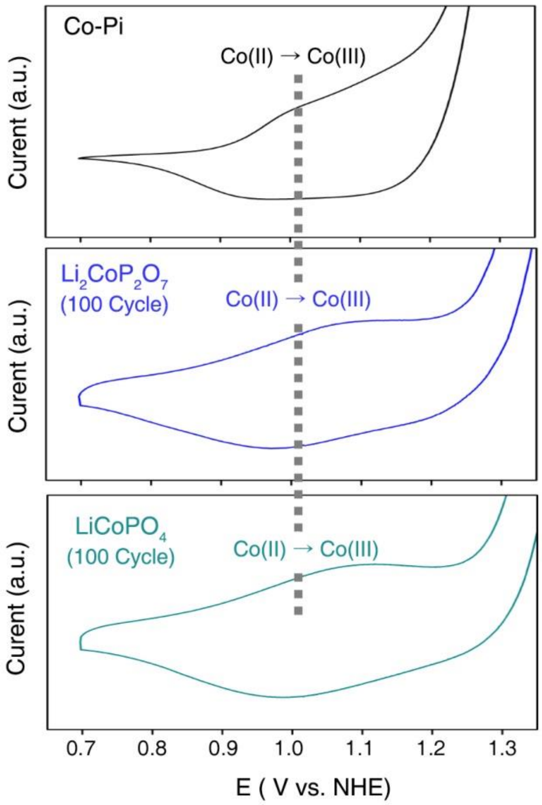

Supplementary Figure 11. Redox features of Co-Pi, $Li_2CoP_2O_7$ and $LiCoPO_4$. Cyclic voltammetry curves for Co-Pi displayed with 100th cycles of $Li_2CoP_2O_7$ and $LiCoPO_4$. Similar Co(II)/Co(III) redox features were observed before the catalytic wave.

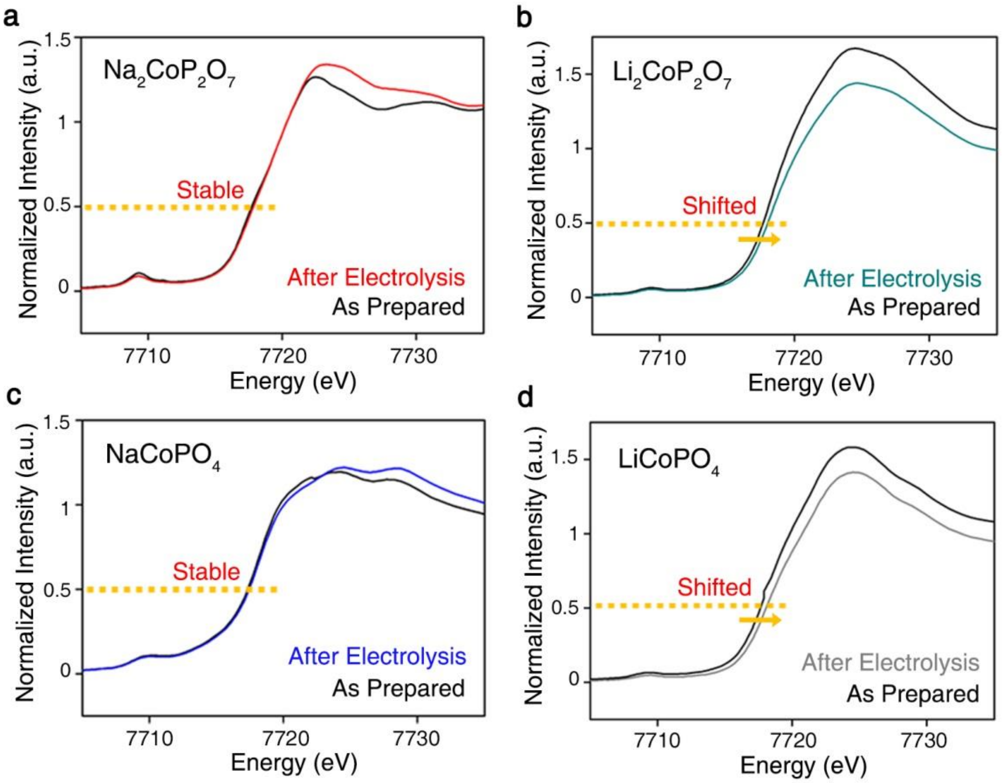

Supplementary Figure 12. *In-situ* XANES spectra of as-prepared and bulk-electrolyzed cobalt phosphate catalysts. *In-situ* XANES spectra corresponding to the Co K-edge of (a) $Na_2CoP_2O_7$, (b) $Li_2CoP_2O_7$, (c) $NaCoPO_4$, and (d) $LiCoPO_4$ before and after bulk electrolysis at an applied potential of 1.5 V vs. NHE for 2 h.

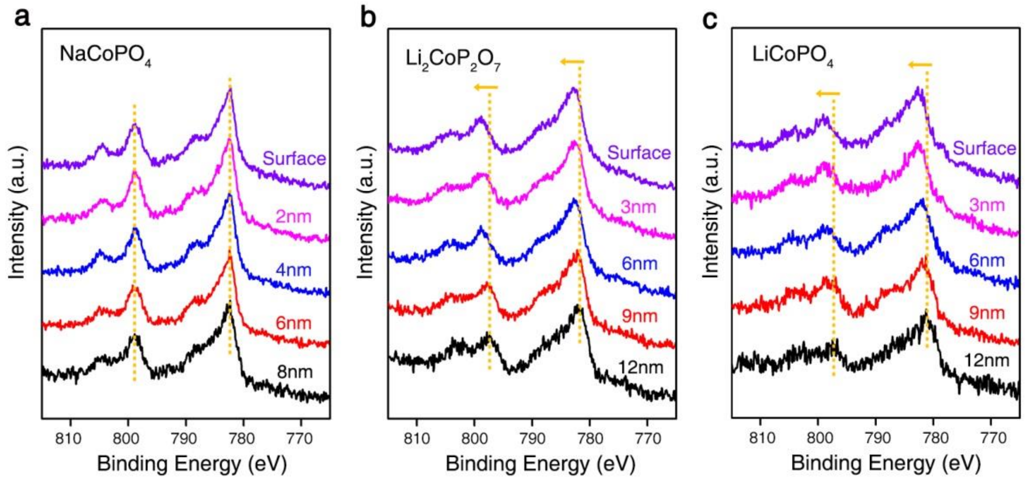

Supplementary Figure 13. XPS spectra of bulk-electrolyzed $NaCoPO_4$, $Li_2CoP_2O_7$ and $LiCoPO_4$. XPS spectra of the Co 2p region of the surface of the bulk-electrolyzed (**a**) $NaCoPO_4$, (**b**) $Li_2CoP_2O_7$, and (**c**) $LiCoPO_4$.

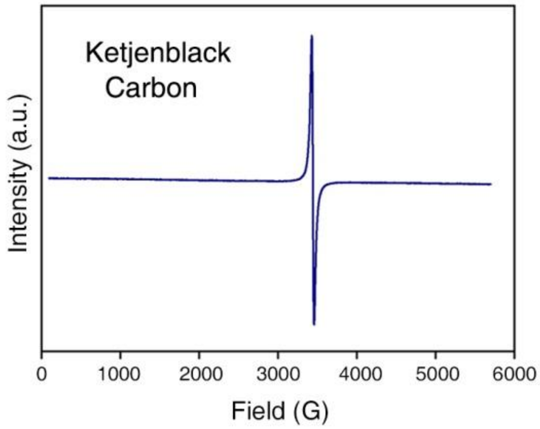

Supplementary Figure 14. CW X-band EPR spectra for Ketjenblack carbon powder. A distinct radical peak around $g_{eff} \approx 2$ can be clearly observed.

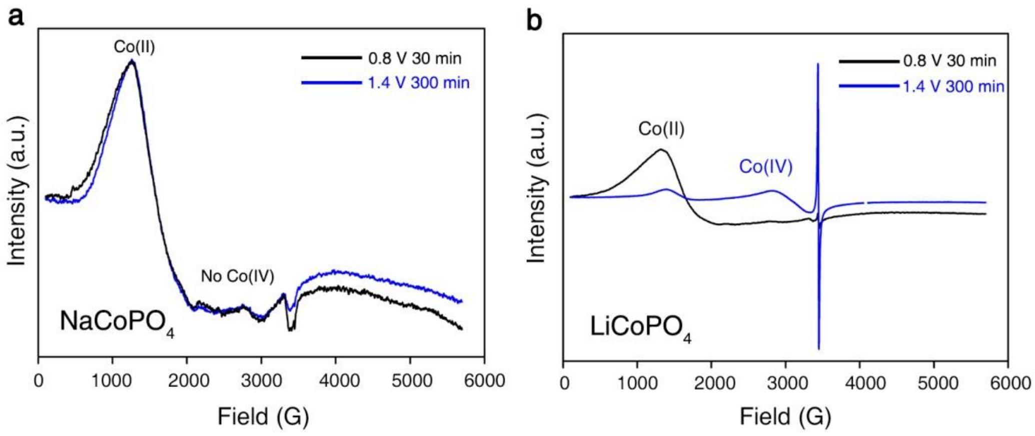

Supplementary Figure 15. CW X-band EPR spectra for $NaCoPO_4$ and $LiCoPO_4$ upon bulk-electrolysis. EPR spectra of $NaCoPO_4$ (**a**) and $LiCoPO_4$ (**b**) upon electrolysis.

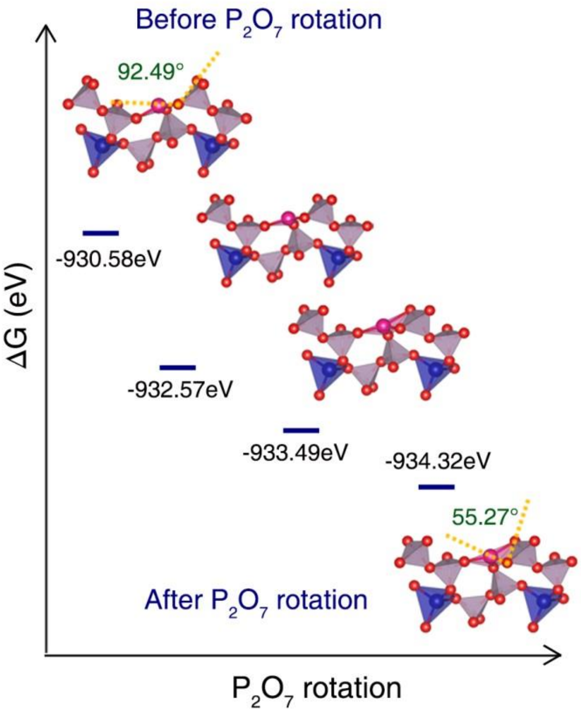

Supplementary Figure 16. Gibbs free energy change during the surface reorganization process. Gibbs free energy reduction after surface reorganization process involving pyrophosphate group rotation is approximately 4 eV per unit cell containing 12 formula $Na_2CoP_2O_7$, clearly demonstrating that overall surface-reorganization process is energetically downhill.

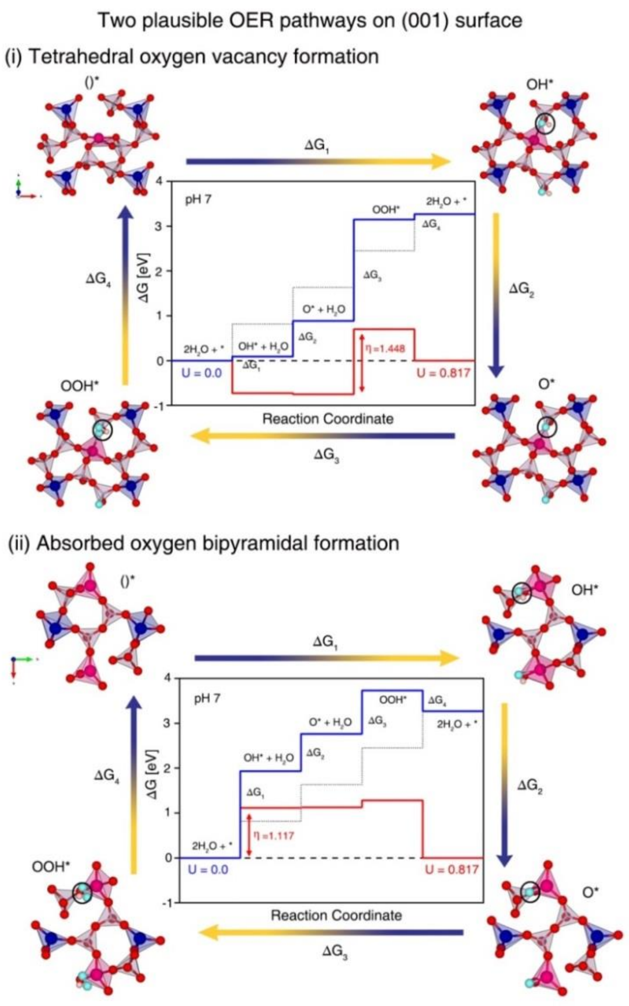

Supplementary Figure 17. DFT calculation for OER mechanism of $Na_2CoP_2O_7$ (001) plane. Schematic of the Gibbs free energy changes for the four elementary steps during the OER on the (001) surface using DFT calculations. The local structure and valence change of the cobalt atoms in the active sites are shown at the corners. The inset shows the free-energy landscape on the (001) surface of $Na_2CoP_2O_7$ compared with an ideal catalyst for pH 7.0. The ideal catalyst, $Na_2CoP_2O_7$ for U=0, and $Na_2CoP_2O_7$ for U=0.817 are depicted by the dashed line, blue line, and red line, respectively.

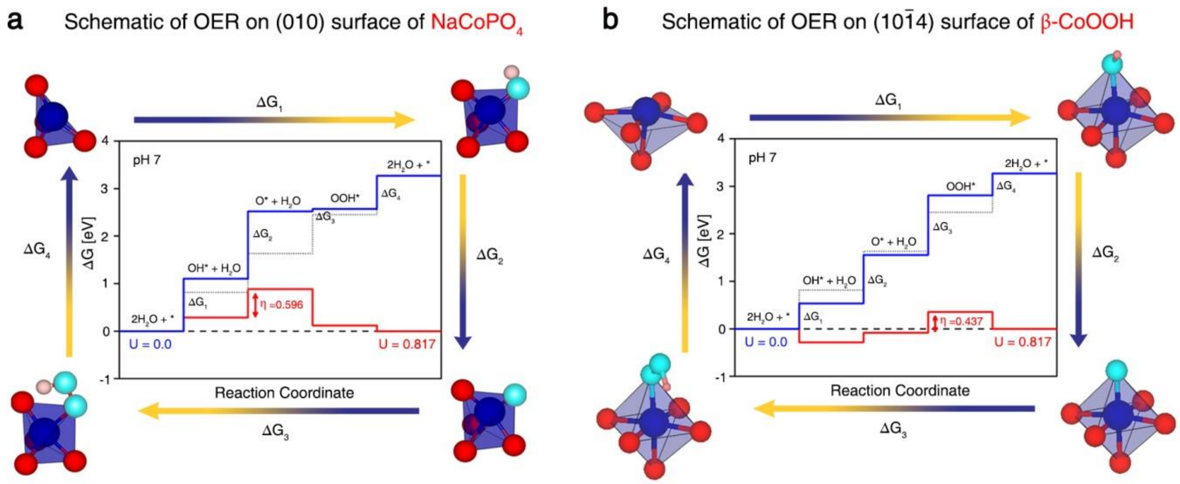

Supplementary Figure 18. DFT calculation for OER mechanism of $NaCoPO_4$ and β-CoOOH. Schematic of the Gibbs free energy changes for the four elementary steps during the OER on the (010) surface of $NaCoPO_4$ (**a**) and the (10$\bar{1}$4) surface of β-CoOOH (**b**) using DFT calculations.

Supplementary Table 1. Structural features of cobalt based oxide / phosphate / pyrophosphate materials

Materials	Symmetry	Co coordination [CoO_x]
	**Cobalt Oxide**	
CoO	Cubic	CoO_6(Oh) edge sharing
Co_2O_3	Trigonal	CoO_6(Oh) edge sharing
Co_3O_4	Cubic	CoO_4 (Td) and CoO_6(Oh)
$LiCoO_2$	Hexagonal	CoO_6(Oh) edge sharing
$NaCoO_2$	Hexagonal	CoO_6(Oh) edge sharing
	**Cobalt Phosphate**	
$Co_3(PO_4)_2$	Monoclinic	CoO_5 (TBP) and CoO_6(Oh) edge sharing
$LiCoPO_4$	Monoclinic	CoO_6(Oh) corner sharing
$NaCoPO_4$	Orthorhombic	CoO_6(Oh) edge sharing
	**Cobalt Pyrophosphate**	
$Co_2P_2O_7$	Monoclinic	CoO_6(Oh) edge sharing
$Li_2CoP_2O_7$	Monoclinic	CoO_5 (TBP) and CoO_6(Oh) edge sharing
$Na_2CoP_2O_7$	Orthorhombic	CoO_4(Td) isolated

Supplementary Table 2. The cell parameters of the four materials were refined by full pattern matching

	a (Å)	b (Å)	c (Å)	V (Å^3)	β (°)
$Na_2CoP_2O_7$	7.704	10.291	15.403	1221.090	90
$Li_2CoP_2O_7$	10.955	9.692	9.761	1014.722	101.763
$NaCoPO_4$	8.883	6.801	5.033	304.076	90
$LiCoPO_4$	10.199	5.924	4.701	284.022	90

Supplementary Table 3. Calculated surface energies of $Na_2CoP_2O_7$

**Orientation**	**Surface Energy (meV Å^{-2})**	**Stoichiometry**
(0 0 1)	0.021	Stoichiometric surface
(1 0 0)	0.057	Non-stoichiometric surface
(1 1 0)	0.062	Non-stoichiometric surface
(2 1 0)	0.067	Non-stoichiometric surface
(1 0 1)	0.016	Stoichiometric surface
(1 1 1)	0.032	Non-stoichiometric surface
(3 -1 0)	0.047	Non-stoichiometric surface

Supplementary Table 4. Calculated DFT energies, zero point energy, and entropy of the water oxidation steps at $Na_2CoP_2O_7$ (101) surface (in eV)

	E^{DFT}	ZPE^{a}	$T\Delta S^{exp\ b}$	E – ZPE + TΔS
*	-948.51	0	0	-948.51
OH*	-958.49	0.37	0	-958.86
O*	-953.41	0.06	0	-953.47
OOH*	-962.95	0.44	0	-963.40
H_2O	-14.03	0.57	0.67 (0.035 bar)	-14.60
H_2	-6.72	0.35	0.403	-7.07

a: Reference 1

b: Reference 2

Supplementary References

1. Bajdich, M., Garcia-Mota, M., Vojvodic, A., Norskov, J.K. & Bell, A. T. Theoretical investigation of the activity of cobalt oxides for the electrochemical oxidation of water. *J. Am. Chem. Soc.* **135**, 13521-13530 (2013).

2. Haynes, W. M. *CRC handbook of chemistry and physics*. CRC press (2013).
